# Anakinra in Sanfilippo syndrome: a phase 1/2 trial

**DOI:** 10.1038/s41591-024-03079-3

**Published:** 2024-06-21

**Authors:** Lynda E. Polgreen, Agnes H. Chen, Youngju Pak, Anna Luzzi, Adolfo Morales Garval, Jonathan Acevedo, Gal Bitan, Michelina Iacovino, Cara O’Neill, Julie B. Eisengart

**Affiliations:** 1 Lundquist Institute for Biomedical Innovation at Harbor-UCLA Medical Center, Torrance, CA USA; 2 Department of Pediatrics, David Geffen School of Medicine, University of California, Los Angeles, Los Angeles, CA USA; 3 Department of Neurology, David Geffen School of Medicine, Brain Research Institute and Molecular Biology Institute University of California, Los Angeles, Los Angeles, CA USA; 4 Cure Sanfilippo Foundation, Columbia, SC USA; 5 Department of Pediatrics, University of Minnesota Medical School, Minneapolis, MN USA

**Keywords:** Phase II trials, Paediatric neurological disorders

## Abstract

Sanfilippo syndrome is a fatal childhood neurodegenerative disorder involving neuroinflammation among multiple pathologies. We hypothesized that anakinra, a recombinant interleukin-1 receptor antagonist, could improve neurobehavioral and functional symptoms owing to its capacity to treat neuroinflammation. This phase 1/2 trial aimed to test the safety, tolerability and effects of anakinra on neurobehavioral, functional and quality-of-life outcomes in patients and their caregivers. The primary outcome was the percent of participants requiring a dose increase at week 8 or week 16. Secondary efficacy outcomes included a multi-domain responder index (MDRI). Twenty-three participants (6–26 years of age) were enrolled. Twenty continued treatment to week 8, and 15 (75%) required an increased dose at week 8 or week 16. There was an improvement in at least one domain in the MDRI in 18 of 21 (86%) at week 8 and in 15 of 16 (94%) at week 36. Seven participants withdrew (intolerability of daily injections and lost to follow-up) before week 36. Adverse events occurred in 22 of 23 (96%) participants, most commonly mild injection site reactions. No serious adverse events were related to anakinra. In conclusion, anakinra was safe and associated with improved neurobehavioral and functional outcomes, supporting continued investigation of anakinra in Sanfilippo syndrome and other mucopolysaccharidoses. ClinicalTrials.gov identifier: NCT04018755.

## Abstract (web summary)

In a phase 1/2 trial, the recombinant interleukin-1 receptor antagonist anankira was safe and associated with clinically meaningful improvements in neurobehavioral and quality-of-life outcomes in patients with Sanfilippo syndrome.

## Main

Sanfilippo syndrome is a fatal childhood neurodegenerative disorder characterized by regression in development, loss of speech, disordered sleep and movement, pain and intensifying neurobehavioral symptoms, such as hyperactivity, agitation, destructiveness, distress/screaming and social disengagement. Symptom onset often begins around age 3–5 years, followed by an unremitting disease course culminating in death in the second or third decade of life^1–4^.

There are no approved therapies for Sanfilippo syndrome. Scientifically referred to as mucopolysaccharidosis type III (MPS III), it is one of a group of seven mucopolysaccharidosis disorders defined by deficiency in lysosomal enzymes critical to degrading glycosaminoglycans (GAGs)^5^. Accumulating GAGs trigger pathological cascades and cellular dysfunction that lead to worsening clinical disease^6^. Enzyme-restorative approaches have been successful in attenuating or halting disease progression in most other forms of MPS^7^, but none has been approved for any of the MPS III subtypes^8^.

As the pursuit of enzyme restoration continues, there is urgency to palliate symptoms that cause suffering for most of the Sanfilippo community. Although clinical endpoints of most MPS III clinical trial programs have focused on neurocognitive decline, recent publications have indicated that pain, disordered sleep and movement and neurobehavioral symptoms are among the most important symptoms to the Sanfilippo community to treat^9,10^. Fortunately, community voicing of these therapeutic needs has been strengthened by a substantial increase in regulatory, scientific and clinical emphasis on these treatment outcomes that are meaningful to patients and families^11,12^.

An expansion of therapeutic approach is needed to treat these symptoms by targeting the pathological cascades triggered by GAG buildup, including neuroinflammation^13–21^, which likely underlies many of the challenging neurobehavioral and functional signs of the disease. Results have been encouraging in MPS III murine models, as central nervous system (CNS) disease and behavior are improved when inflammation is successfully decreased, such as by increasing interleukin-1 receptor antagonist (IL-1Ra) via hematopoietic stem cell gene therapy or knocking out the IL-1 receptor^13,14,16,18,19^.

A critical opportunity to apply this alternative approach in humans is afforded by repurposing anakinra (Kineret, Swedish Orphan Biovitrum AB), a recombinant human IL-1Ra that treats other neuroinflammatory diseases^22–26^. Anakinra is approved by the US Food & Drug Administration (FDA) for treatment of cryopyrin-associated periodic syndromes (CAPS), neonatal-onset multi-inflammatory disease (NOMID), deficiency of interleukin-1 receptor antagonist (DIRA) and rheumatoid arthritis. We hypothesized that anakinra could improve neurobehavioral and functional symptoms owing to its capacity to treat both central and peripheral inflammation triggered by GAG accumulation. Here we present the final report on a phase 1/2 study of anakinra in Sanfilippo syndrome, which tested the safety, tolerability and effects on neurobehavioral, functional and quality-of-life outcomes in patients and their caregivers.

## Results

### Patient disposition

**Fig. 1:**
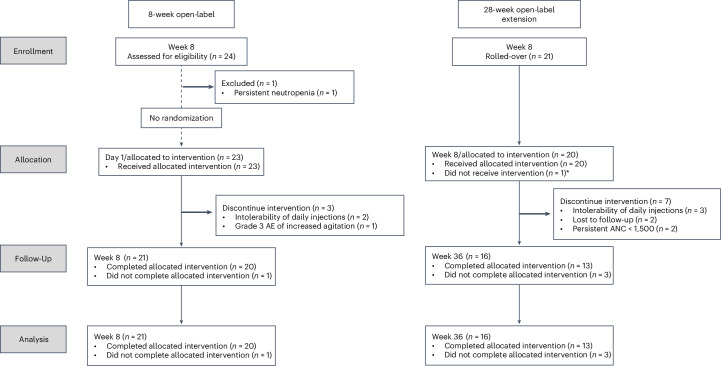
CONSORT flowchart. Anakinra treatment for the 8-week open label from day 1 to week 8 with 28-week open-label extension. Treatment occurred from day 1 to week 36. Observation occurred for 8 weeks before day 1 and for another 8 weeks starting at week 36 when treatment was stopped. The primary enrollment target was 20 on treatment at week 8. *n* = 3 patients stopped treatment but continued the study by completing assessments through week 44: one before week 8 (stopped treatment owing to a grade 3 AE of increased agitation) and two between week 8 and week 36 (stopped treatment owing to persistent ANC < 1,500 cells per microliter).

This was a phase 1/2, 8-week open-label study, followed by a 28-week open-label extension, all of which were preceded and followed by 8-week observational periods (NCT04018755; see protocol in the Supplementary Information). Twenty-four participants (12 males and 12 females) aged 6–26 years were screened, and 23 were enrolled (Fig. 1). One participant was excluded owing to persistent neutropenia during the screening process. Once enrollment was complete, potential participants more than doubling the study size (*n* = 27) chose to join a waitlist in the event the study was expanded or extended or future trials were to be developed. Baseline characteristics of enrolled participants are shown in Table 1. Most participants were categorized by their parents/caregivers as White (88%) and not Hispanic (88%). Although speaking English was not an inclusion criterion, all caregivers who completed surveys spoke and read English fluently.

**Table 1:** Patient characteristics at baseline (*n* = 24)

Age (years)	
Mean ± s.d.	10.6 ± 4.2
Median	9.7
Range	6.3–26.1
Race – no. (%)^a^	
White	21 (88)
Black	1 (4)
Asian	1 (4)
Other	1 (4)
Ethnicity – no. (%)^a^	
Hispanic	3 (13)
Non-Hispanic	21 (88)
Sex – no. (%)	
Female	12 (50)
Male	12 (50)
MPS type – no. (%)	
MPS IIIA	20 (83)
MPS IIIB	3 (13)
MPS IIIC	1 (4)
Past experimental therapy – no. (%)^b^	
Gene therapy	3 (13)
Enzyme replacement therapy	3 (13)
Vineland Adaptive Behavior Scales^41^, Third Edition, Adaptive Behavior Composite^c^	
Mean ± s.d.	42
Median	37
Range	25–69

^a^ Race and ethnic group were reported by the participant’s parent/caregiver.

^b^ Participants were not currently enrolled in another ongoing therapeutic clinical trial.

^c^ Normative population mean ± s.d. is 100 ± 15. Scores within one standard deviation of the mean—that is, 85–115—are in the ‘average’ range, whereas 70–84 is below average, and less than 70 is impaired^34^.

Five participants withdrew owing to intolerability of daily subcutaneous (SC) injections (two before week 8 and three between weeks 16 and 36); two participants were lost to follow-up; and three participants were withdrawn by the principal investigator owing to adverse events (AEs), one for a grade 3 AE and two for persistent neutropenia (<1,500 cells per microliter), withdrawn before the decision to lower the study neutropenia threshold to 1,200 cells per microliter. Reasons for intolerability were reported as difficulty keeping the participant still for injections, particularly when only one caregiver was routinely available; participant distress in anticipation or delivery of injections; and overall burden of route of administration.

### Primary outcome(s)

#### Dosing

Anakinra was started on day 1 at an SC daily dose of 100 mg. Dose escalations were made as follows. Either at week 8 or at week 16 (if not changed at week 8), the daily dose of anakinra was increased to 200 mg SC daily if there was not an improvement from day 1 to week 8 (or week 16) of more than the predefined minimal clinically important difference^27,28^ (MCID—that is, the smallest improvement considered worthwhile to a patient) in the two outcomes chosen by the parent/caregiver to be ‘most bothersome’ at screening. Of the 20 participants who continued treatment to week 8, 12 (60%) required an increased dose of anakinra from 100 mg SC daily to 200 mg SC daily at week 8, owing to lack of improvement; three additional participants had their dose increased at week 16. No statistically significant differences were observed between those who dose escalated and those who did not in age, sex, race or ethnicity, nor in efficacy assessments at baseline.

#### Safety

Mean treatment duration was 187 ± 92 d, and doses missed were, on average, 5.2 ± 6.6 doses. Table 2 summarizes AEs that occurred in at least 5% of participants during treatment with anakinra, and Extended Data Table 1 summarizes all AEs occurring during the overall study. Of the 23 participants who received at least one dose of anakinra, 22 (96%) reported at least one AE during the study; the most common AEs were injection site reactions, most frequently erythema and swelling, reported in 74% and 43%, respectively. There were no unexpected AEs. Three serious adverse events (SAEs) occurred, none of which was determined to be related to anakinra exposure: two during treatment with anakinra (viral pneumonia and injury (muscle laceration)) and one after treatment (upper gastrointestinal hemorrhage).

**Table 2:** AEs that occurred in at least 5% of participants

AE	On treatment (*n* = 23)				After treatment^a^ (*n* = 16)			
	Incidence		Event rate		Incidence		Event rate	
	*n*	%	*n*	Rate	*n*	%	*n*	Rate
Participant with any SAE^b^	2	9%	2	0.09	1	4%	1	0.04
Participant with any AE	22	96%	408	17.74	15	65%	52	2.26
Injection site AEs								
Injection site reaction (any)	18	78%	326	14.17				
Injection site erythema	17	74%	165	7.17				
Injection site swelling	10	43%	52	2.26				
Injection site bruising	5	22%	41	1.78				
Injection site itching	3	13%	65	2.83				
Injection site bleeding	3	13%	3	0.13				
Non-injection site AEs								
Upper respiratory infection	7	30%	8	0.35	4	17%	4	0.17
Constipation	4	17%	5	0.22	4	17%	4	0.17
Agitation	4	17%	4	0.17	2	9%	2	0.09
Neutropenia	6	26%	9	0.39	1	4%	1	0.04
COVID-19—mild symptoms	5	22%	5	0.22	0	0%	0	0.00
Diarrhea	4	17%	4	0.17	1	4%	1	0.04
Acute otitis media	2	9%	2	0.09	2	9%	3	0.13
Thrombocytopenia	4	17%	4	0.17	1	4%	1	0.04
Rash	1	4%	1	0.04	2	9%	2	0.09
Seizures	1	4%	1	0.04	2	9%	3	0.13
Stiffness in arms and legs	1	4%	1	0.04	2	9%	2	0.09
Bronchitis	2	9%	2	0.09	0	0%	0	0.00

^a^ AEs occurring either after early drug discontinuation for participants who continued in the study off treatment or after discontinuation of treatment at week 36 to week 48. Complete reporting of AEs is available in Extended Data Table 1

^b^ No treatment-related SAEs.

### Secondary outcomes

**Fig. 2:**
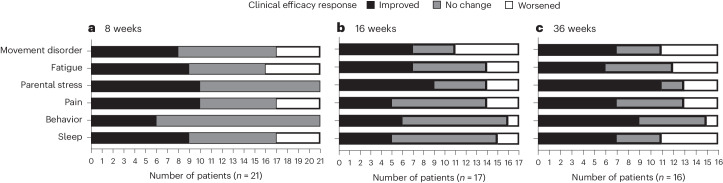
MDRI: comparison of change during 8 weeks (a), 16 weeks (b) and 36 weeks (c) of treatment to MCID. A change during treatment with anakinra relative to the MCID was used to define ‘improved’, ‘no change’ and ‘worsened’. MCID definitions can be found in Methods, ‘Secondary outcomes’.

Secondary outcomes, to evaluate efficacy, were selected to account for the heterogeneity of MPS III symptoms, informed by the caregiver community^9,10^ and consensus recommendations on Sanfilippo trial design^29,30^. A multi-domain responder index (MDRI) and an individual clinical response (ICR) were used to capture heterogeneity in treatment response, similar to what was used previously^31,32^. Most participants showed improvement beginning at 8 weeks of treatment as demonstrated by the MDRI approach for measuring heterogeneous treatment effects (Fig. 2). Specifically, 18 of 21 (86%) participants improved on one or more of the six outcomes included in the MDRI at week 8, and 15 of 16 (94%) participants improved on one or more of the six outcomes at week 36. After 8 weeks of treatment, improvement of more than the MCID was most common for parenting stress (48%), measured by the Autism Parenting Stress Index (APSI), and pain (48%), measured by the Non-Communicating Children’s Pain Checklist–Revised (NCCPC-R). After 36 weeks of treatment, improvement was still most common for parenting stress (69%), but the second-most common improvement was behavioral symptoms (56%), measured by the total averaged score on the Sanfilippo Behavior Rating Score (SBRS), followed by pain (44%).

**Extended Data Fig. 1:**
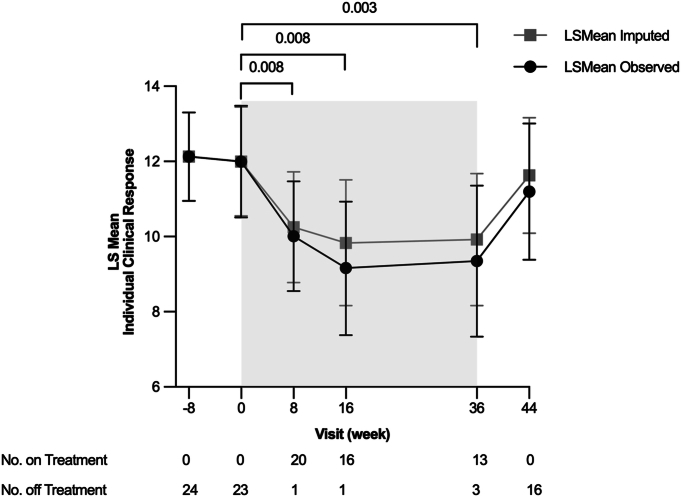
Change in the Individual Clinical Response (ICR) before, during and after treatment with anakinra. Treatment period indicated by grey box (Week Zero to Week 36). The black line graph with circle symbols shows the observed LSMeans with 95% confidence intervals for patients with available data (observed) over time. The imputed LSMeans for the prespecified sensitivity analysis are plotted as grey lines with square symbols. A return of symptoms after eight weeks off treatment, seen by the upward slope to Week 44, suggests the reduction of symptoms from Weeks Zero to 36 were not part of the natural disease course. There was no statistically significant difference in ICR between Week 44 and Day One. All statistical tests were two-sided, including the Wald test with 23 degrees of freedom from the mixed model. No adjustment was made for the multiple comparison.

Based on the estimated least-squares means, there was an improvement in the ICR from day 1 to week 8 (−2.0, 95% confidence interval (CI): −3.4, −0.6), from day 1 to week 16 (−2.8, 95% CI: −4.9, −0.8) and from day 1 to week 36 (−2.7, 95% CI: −4.3, −1.0). A return of symptoms after 8 weeks without treatment is demonstrated in the upward slope (that is, increased severity) between week 36 and week 44 (Extended Data Fig. 1). There was no difference in ICR between the last day of the pre-dosing observational period and the last day of the post-dosing observational period (0.93, 95% CI: −1.18, 3.04).

**Fig. 3:**
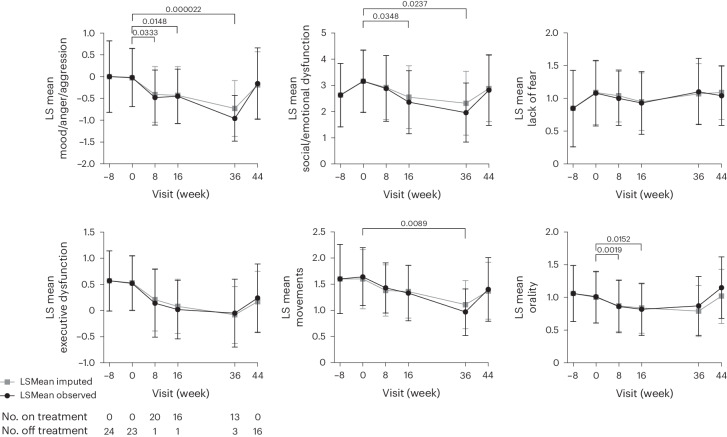
Change in the SBRS individual clusters and domains before, during and after treatment with anakinra. The treatment period was from week 0 to week 36. The black line graph with circle symbols shows the observed least-squares means with 95% CIs for patients with available data (observed) over time. The imputed least-squares means for the pre-specified sensitivity analysis are plotted as gray lines with square symbols. *P* values are for change in observed least-squares means from day 1. A return of symptoms after 8 weeks off treatment, seen by the upward slope to week 44, suggests that the reduction of symptoms from week 0 to week 36 was not part of the natural disease course. There was no statistically significant difference in SBRS scores between week 44 and day 1. All statistical tests were two-sided, including the Wald test with 23 degrees of freedom from the mixed model. No adjustment was made for multiple comparisons. LSMean, least-squares mean.

Estimated least-squares means and 95% CIs before, during and after treatment with anakinra in the SBRS clusters (Movements, Social/Emotional Dysfunction, Lack of Fear and Executive Dysfunction) and domains (Orality and Mood/Anger/Aggression) are shown in Fig. 3 and Extended Data Table 2, and descriptive statistics are shown in Extended Data Table 3. There was a positive effect of treatment in all clusters and domains of the SBRS except for the Lack of Fear and Executive Dysfunction clusters, where there was no statistically significant response to treatment with anakinra. On all clusters and domains except for the Lack of Fear cluster, a return of symptoms off treatment is depicted in the upward slope between week 36 and week 44, indicating rebounding frequency of symptoms (Fig. 3). There was no difference in any of the SBRS clusters or domains between the last day of the pre-dosing observational period and the last day of the post-dosing observational period (Extended Data Table 2).

The least-squares mean difference in parenting stress measured by the APSI from day 1 to week 16 was −2.9 (95% CI: −5.2, −0.7) and from day 1 to week 36 was −3.8 (95% CI: −6.8, −0.8). No significant changes were observed in least-squares means for the Child Sleep Health Questionnaire (CSHQ), the PROMIS Fatigue–Parent Proxy Custom Short Form, the disordered movement 7-d log or the NCCPC-R. Least-squares means for all the MDRI outcomes are detailed in Extended Data Table 4, and descriptive statistics are provided in Extended Data Table 5.

### Post hoc analyses

**Fig. 4:**
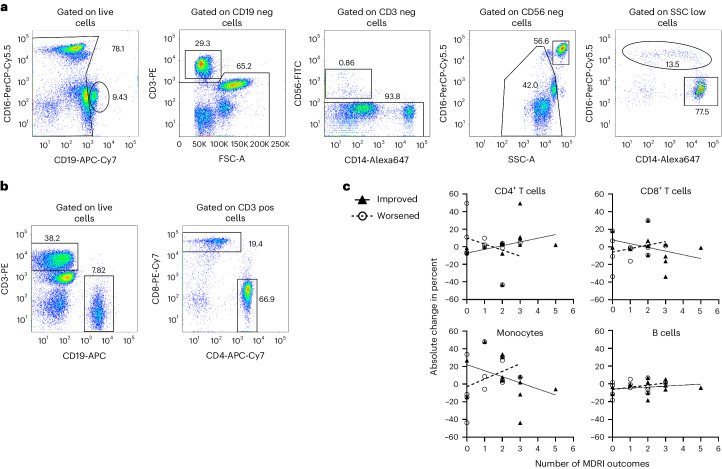
Immunophenotype changes compared to the MDRI. **a**, Flow cytometry plot of whole blood stained with specific antibodies to identify monocytes. Whole blood cells were gated on live cells and then sub-gated on CD19^−^ cells (to exclude B cells), CD3^−^ cells (to exclude T cells) and CD56 cells (to exclude NK cells). Cells that were not bright CD16 (neutrophils) in the side scatter plot (SSC) plot were identified as monocytes. These cells expressed CD14 and CD16. **b**, B lymphocytes were identified as CD19^+^ cells and CD3^−^ cells. T lymphocytes CD4 and CD8 were sub-gated from CD3^+^ cells. **c**, Trends for changes in CD4^+^ T cells (for example, T helper and T regulatory cells), CD8^+^ T cells (for example, cytotoxic T cells), monocytes and B cells over 16 weeks of treatment with anakinra, compared to the MDRI. The number of MDRI surveys with a treatment response that was either improved (black triangles/solid line) or worsened (open circles/dashed line), as defined by the MCID, is plotted with immunophenotype data as percent of the cell population. The *y* axis is absolute change in percent of the cell population. Positive numbers are an increase in cell population, and negative numbers are a decrease in cell population. An increase in CD4^+^ T cells with a decrease in CD8^+^ T cells and a decrease in monocytes is suggestive of a less pro-inflammatory environment. This less pro-inflammatory environment is the desired change with therapy. These changes coincide with a higher number of improvements on the MDRI outcomes. Anakinra is not expected to have a significant effect on B cells. The lack of B cell change and lack of relationship with MDRI outcomes may increase confidence in the findings. * Spearman’s rank-based correlation coefficient *P* < 0.05. SSC-A, side scatter area. Source data

Post hoc analysis to investigate a relationship of the biochemical effects of anakinra with the clinical effects found a statistically significant correlation between an increase in CD4^+^ T cells and a higher number of improved outcomes in the MDRI (Fig. 4). Although correlations with other cell types in this small sample did not reach statistical significance, in general, the less pro-inflammatory the environment (the desired effect—that is, an increase in CD4^+^ T cells and decreases in CD8^+^ T cells and in monocytes), the more the MDRI outcomes showed improvements (Fig. 4). In contrast, MDRI outcomes that showed worsening were associated with more pro-inflammatory markers—that is, decrease in CD4^+^ T cells and increases in CD8^+^ T cells and in monocytes.

## Discussion

Curative therapies for Sanfilippo syndrome are in development, but the lack of any approved disease-modifying treatment creates urgency to palliate the challenging symptoms of this largest affected segment of the MPS population. Discovery of the key pathologic role of neuroinflammation in MPS has led to critical opportunities to examine the potential for attainable relief on a shorter timeline by reducing inflammation and its clinical correlates^13^. This study of anakinra in Sanfilippo syndrome is, to our knowledge, the first in-human study to repurpose an FDA-approved, blood-brain-barrier-crossing, anti-inflammatory therapy to target CNS inflammation in MPS. Aligned with the purpose of a phase 1/2 trial, our findings indicate that anakinra is safe for further investigation while curative therapies are in development. Furthermore, findings show clinically meaningful improvements in various neurobehavioral and quality-of-life outcomes, suggesting that an anti-inflammatory approach might prove to be symptom modifying for Sanfilippo syndrome.

No new safety concerns were identified. All AEs were mild or moderate, and no SAEs were considered to be related to anakinra. However, a high number of participants withdrew, primarily owing to the challenge of administering daily SC injections. Specifically, two caregivers were often needed to hold the participant and administer the injection, due to the highly physical and dysregulated behaviors of Sanfilippo syndrome. In single-caregiver households, the challenge of daily injections was considerable.

The design of this trial and the choice of endpoints were founded on consensus guidelines for MPS III (ref. ^29^), patient/caregiver input^9,10^ and FDA guidance to include patient and community input when designing trials^12,33^. Unlike enzyme-restorative trials for MPS III, this study differed in a few key ways: (1) inclusion of patients with advanced disease and across all MPS III disease subtypes; (2) incorporation of the Sanfilippo community perspective in the design and execution of the trial; and (3) use of a composite measure to capture heterogenous treatment effects. The composite measure in this trial was an MDRI, an approach that evaluates a broad, heterogeneous array of clinical disease manifestations to understand fully the treatment effects^32^. The MCID enables interpretation of the meaningfulness of individual numeric changes on a variety of outcome measures. The patient perspective was incorporated at study conception, trial design and through study completion via partnership with the one of the largest Sanfilippo syndrome patient advocacy groups, which has a global reach, and further involvement of its chief science officer as a co-investigator. This close partnership allowed the study team deeper insights into the community’s priorities. Recognizing the need to capture the effect of treatment upon individuals’ most meaningful symptom targets, and that those targets may vary in importance from patient to patient, we developed an individual clinical response measure. This newly developed measure allowed caregivers to choose outcomes that mattered the most to them, and, thus, we could collect more personalized data on longitudinal treatment impacts with inherent meaningfulness.

The severity of disease in our cohort was by design to ensure improved representation of the substantial segment of the Sanfilippo community who have been excluded from enzyme-restorative trials. It further ensured that this study would not compete with potentially curative trials that target neurocognitive decline. Targeting neurocognition is not feasible in our study population as this aspect of function is often very low and minimally changing past the age of 6 years in most MPS III types^34–36^. Although the historical focus on neurocognition has been key to revealing whether enzyme-restorative trials modify the course of decline^34,36^, this focus does not address many of the symptoms prioritized by the patient community^9,10^, which was the emphasis of the present study.

The SBRS was included because it has been validated in Sanfilippo samples^37^, correlated with biomarkers^37–39^ and recommended for trial use at two international meetings^29,30^. The SBRS identified a positive effect of anakinra in all clusters and domains, except for the Lack of Fear cluster, which was anticipated, and Executive Dysfunction. Given Sanfilippo-related atrophy of the amygdala^37–39^, a subcortical structure linked to fear, we did not expect decreasing inflammation to have a substantial effect on the SBRS Fear cluster. These non-findings in the Lack of Fear domain may strengthen the validity of this study’s positive SBRS findings on the other domains and clusters.

The natural histories of this study’s endpoints have not been reported as plentifully as neurocognition or adaptive behavior in Sanfilippo syndrome. Therefore, we examined these endpoints in untreated individuals during the observational periods, balancing the duration of observation with the urgency to relieve symptoms. An important consideration was the natural reduction in symptoms associated with neurodegeneration: neurobehavioral manifestations lessen as the disease progresses^40^, due to continual loss in multiple functions coincident with neurodegeneration. Thus, we remained mindful that the appearance of improvement in symptom intensity can be a sign of progressing disease, which we did not want to mistake for therapeutic benefit. After cessation of anakinra, target symptoms rebounded to pre-dosing severity/frequency, suggesting that the reduction in symptoms during the treatment period was not explained by disease progression. Across domains, upward trajectories of symptoms (that is, worsening) when the patients come off treatment to the end of untreated observation increase optimism that the period of improvement is related to anakinra.

This study has several limitations. Although it is common that a placebo is not part of a phase 1/2 study, our decision not to include placebo in this early investigation was also an ethical one, as anakinra is given as a daily SC injection. This does, however, mean that our results need to be interpreted with caution. For example, the ICR has clinical importance because it was developed to prioritize caregiver concerns, but it is prone to effect size inflation, especially in this non-controlled study. We also recognize that interpretation of efficacy is limited by caregiver report in an unblinded trial. However, it was the only feasible method to assess the community-specified target symptoms^9,10^ because affected children in this study’s age range typically have severely impaired communication and cognition. Furthermore, post hoc analysis did show a correlation of a less inflammatory immunophenotype aligning with caregiver report of benefits. As a final point, although efforts were made to use assessment tools that addressed the priority symptoms of Sanfilippo syndrome, even these methods could not fully capture all types of benefits described by caregivers via investigator patient history interviews, spontaneous reporting throughout the trial or informal exit interviews. Planned qualitative family interviews were highlighted as an important need in the design of a future trial.

The results from this phase 1/2 study provide initial evidence that targeting inflammation through the use of anakinra is safe and may improve neurobehavioral symptoms and meaningful aspects of the lived experience of patients and families affected by Sanfilippo syndrome. Findings may have broader implications for targeting central and peripheral inflammation in other MPS types as well as other neurodegenerative diseases.

## Methods

### Trial design and oversight

This study was conducted from January 2020 through March 2023 at a single site. The design was developed in partnership with one of the largest Sanfilippo syndrome patient advocacy groups, the Cure Sanfilippo Foundation, which has been involved with multiple international research initiatives. Its chief science officer (author C.O.) served as a co-investigator, enabling integration of patient perspectives in design and study implementation throughout.

### Ethics

An independent external data and safety monitor reviewed all data. The trial was performed according to the Declaration of Helsinki. Independent ethics committee approval was obtained from the John F. Wolf, M.D. Human Subjects Committee at the Lundquist Institute for Biomedical Innovation at the Harbor-UCLA Medical Center. Participants’ legal guardians provided written informed consent. No participant was cognitively able to provide consent or assent.

### Study enrollment

Participants were recruited nationwide with the help of the Cure Sanfilippo Foundation and publicly posted study announcements. Participants were screened in the order that they contacted the study. Participant sex was collected from the parent/caregiver. Gender data were not collected. This is due to the severe cognitive deficits in our study population such that they are unable to self-report sex or gender. Sex and/or gender were not considered in the study design. Full inclusion and exclusion criteria are as follows:

#### Inclusion criteria

- MPS III diagnosis confirmed by genetic testing.
- ≥4 years old.
- Patient or parent/legal guardian were able and willing to provide informed consent. For patients 7–17 years of age, assent was provided when cognitively possible.
- If on genistein, a stable dose for 6 months was required before enrollment.
- If on melatonin or other sleep medications, stable doses were required for the past 3 months.
- Two of the following criteria were met:
  1. CSHQ Total score ≥ 41.
  2. SBRS Cluster or Domain score ≥ −2 s.d. of mean for age group.
  3. The presence of significant MPS III-related CNS impairment or behavioral disturbances.
  4. NCCPC-R Total Score ≥ 7.
  5. Seizure disorder thought to be due to MPS III–related disease changes, requiring use of regular medication.
  6. Presence of a movement disorder.

OR one of the following criteria was met:

1. Previous participation in a gene/cell therapy or enzyme-restorative clinical trial.
2. Previous exclusion from a gene/cell therapy or enzyme-restorative clinical trial.
3. Functional age as measured by the Vineland Adaptive Behavior Scales (Second or Third Edition^41,42^) was ≤0.5 chronological age. The Vineland was recommended at two international consensus conferences^30,43^ to assess daily functioning in MPS and has been shown to be an appropriate measure of functional level in MPS III (refs. ^1,35,36,44^).

#### Exclusion criteria

- Currently enrolled in another clinical treatment trial.
- Previous or current treatment with anakinra, canakinumab or any other IL-1 inhibitor.
- Use of any of the following therapies before enrollment:
  - Narcotic analgesics (within 24 h)
  - Tocilizumab, dapsone or mycophenolate mofetil (within 3 weeks)
  - Etanercept, leflunomide, thalidomide or cyclosporine or intraarticular, intramuscular, intravenous or oral administration of glucocorticoids (within 4 weeks)
  - Intravenous immunoglobulin (IVIG), adalimumab or methotrexate (within 8 weeks).
  - Infliximab, 6-mercaptopurine, azathioprine, cyclophosphamide or chlorambucil (within 12 weeks)
  - Rituximab (within 26 weeks)
- Live vaccines within 1 month before enrollment.
- Known presence or suspicion of active, chronic or recurrent serious bacterial, fungal or viral infections, including tuberculosis (TB), HIV infection or hepatitis B or C infection.
- Clinical evidence of liver disease or liver injury as indicated by the presence of abnormal liver tests.
  - AST or ALT > 5× upper limit of normal (ULN) or
  - AST or ALT > 3× ULN accompanied by elevated bilirubin >2× ULN.
- Severe renal function impairment (estimated creatinine clearance < 30 ml min^−1^ 1.73 m^−2^).
- Neutropenia (defined as absolute neutrophil count (ANC) < 1,200 cells per microliter).
- History of malignancy.
- Hypersensitivity to *Escherichia coli*–derived proteins or any components of anakinra.
- Pregnant or lactating women.
- Current active infection.
- History of serious opportunistic infection (for example, bacterial (Legionella and Listeria); TB; invasive fungal infections; or viral, parasitic and other opportunistic infections).
- Positive TB skin test, positive QuantiFERON-TB Gold test, positive chest X-ray or a recent exposure to TB.
- Live vaccine exposure that would be required to occur during the study.
- Any other social or medical condition that the investigator thinks would pose a substantial hazard to the participant if the investigational therapy were initiated or be detrimental to the study.

### Dosing

Anakinra SC was administered at 100 mg or 200 mg SC once daily. The starting dose (100 mg SC once daily) is the dose level recommended for adult rheumatoid arthritis. The dose escalation was within the range of the recommended dose in NOMID. Non-weight-based dosing was based on pharmacokinetic data in 22 pediatric patients with systemic-onset juvenile idiopathic arthritis in the age range of 2–17 years^45^.

Dose was increased to 200 mg SC once daily, with a maximum dose limit of 8 mg kg^−1^ d^−1^ at week +8 or week +16, if the change in at least one of the two most bothersome outcomes selected by the parents/guardians at day 1 had not improved by ≥ the MCID. The exception was the disordered movement 7-d log, which did not have a calculatable MCID until the dataset was complete; therefore, dose was increased if there was worsening or no change in either the Duration or Severity as described below. After increase at week +8, if the MCID was again not achieved after eight sustained weeks on the maximum trial dose of 200 mg (maximum dose 8 mg kg^−1^ d^−1^), treatment with anakinra could be discontinued after a review and discussion between the study principal investigator and the participant’s parent(s) of their child’s individual results from all outcome measures.

For example:

- At week +8, if a participant had not improved in the chosen items, then the dose was increased to 200 mg SC daily.
- At week +8, if a subject had improved, then the dose stayed at 100 mg SC daily. However, if this individual then had worsening of the two most bothersome outcomes from week +8 to week +16, after improving from day 1 to week +8, the dose was increased to 200 mg SC daily at that point (that is, increase at week +16 after not increasing at week +8).

Dose was decreased by 50 mg SC once daily at any time throughout the study if a participant developed any of the following:

- Neutropenia <1,200 cells per microliter persistent for ≥2 weeks (decreased from 1,500 cells per microliter on 11 January 2021)
- Thrombocytopenia <50 × 10^9^ platelets per liter persistent for ≥2 weeks
- Mild/moderate hypersensitivity reactions, including urticaria, rash and pruritis
- Common Terminology Criteria for Adverse Events grade 3 AE

Anakinra was stopped if the AE above did not resolve within 2 weeks of decreasing the dose by 50 mg SC once daily. Anakinra was restarted at 0.5 times the last dose administered once the AE was resolved. If the AE recurred with restarting anakinra, it was discontinued, and the participant was monitored per protocol.

### Outcomes

#### Primary outcomes

The primary safety and tolerability endpoint (phase 1) was defined as the occurrence of AEs and SAEs. The primary dosing (phase 2) endpoint was the percent of participants who required an increase in anakinra dose from 100 mg SC daily to 200 mg SC daily at week 8 or week 16.

### Secondary outcomes

#### MDRI

The MDRI was composed of an SBRS and standardized ratings of sleep habits (CSHQ), fatigue (PROMIS–Fatigue Parent Proxy Custom Short Form), pain (NCCPC-R) and parenting stress (APSI) as well as parent tracking of disordered movement (disordered movement 7-d log). The MCID, used to determine improvement (>1 MCID in direction of improvement), worsening (>1 MCID in direction of worsening) or no change (<1 MCID in either direction) on the MDRI, was defined for each outcome based on previous reports in the literature when available or the change during the first 8-week observation period when not available (that is, disordered movement log).

Additional secondary outcomes were mean change in each survey included in the MDRI, described below, followed by a description of the ICR.

#### Sleep

##### CSHQ (paper form)

The CSHQ is a parent questionnaire that has been used in many studies to examine both behavioral-based and medical-based sleep problems in children. It yields a total score and eight subscale scores: (1) Bedtime Resistance, (2) Sleep Onset Delay, (3) Sleep Duration, (4) Sleep Anxiety, (5) Night Wakings, (6) Parasomnias, (7) Sleep-Disordered Breathing and (8) Daytime Sleepiness. It has been validated in multiple groups, including community children and children diagnosed with sleep disorders, development delay and/or autism^46,47^.

Scoring: Higher scores indicate more significant and frequent symptoms. Each item is scored on a scale of 1–3, and a total sleep disturbances score may range from 33 to 99.

MCID = 3.2 based on 469 children aged 4–10 years^46^.

#### Behavior

##### SBRS (paper form)

The SBRS is a 68-item questionnaire developed to assess the behavioral phenotype of children with MPS III and its progression over time^37,48^. The SBRS has been validated in Sanfilippo samples^37^ and correlated with biomarkers^37–39^. There are 15 ‘domain scales’ that rate the frequency of symptoms related to orality, movement/activity, attention/self-control, emotional function and social interaction. In addition, 12 of the 15 domain scales are grouped into four abnormality clusters: Movements, Lack of Fear, Social/Emotional Dysfunction and Executive Dysfunction. Two domain scales are recommended by the test developers to be analyzed separately: Orality and Mood/Anger/Aggression.

Scoring: Higher scores indicate higher frequency of symptoms. Each item is scored on a scale of 0–6. A mean score is calculated for each SBRS domain and cluster, and then an average is taken for the SBRS Total score. The mean scores were standardized using the mean and standard deviation from a cohort of patients with MPS III, ages 81–220 months^48^. This reference cohort was chosen to best match the age distribution of our participants.

MCID (SBRS Total mean score only) = 0.57 based on 18 children with Sanfilippo syndrome aged 6–23 years^38^.

#### Pain

##### NCCPC-R (paper form)

The NCCPC-R is validated for measuring pain in children with severe cognitive impairments^49,50^. It includes seven scales that measure vocal, social, facial, activity, body and limbs, physiological and eating/sleeping indicators of pain. A combined total score for pain was calculated.

Scoring: Higher scores indicate greater pain. Each item is scored on a scale of 0–3, and a total pain score may range from 0 to 99.

MCID = 4.6 based on 57 non-verbal children aged 3–18 years^50^.

#### Parent fatigue

##### PROMIS Fatigue–Parent Proxy Custom Short Form (paper form)

Per PROMIS guidance, we selected the 10 most relevant questions for parents of children with MPS III from the PROMIS Parent Proxy Fatigue item bank to make a customized PROMIS Fatigue–Parent Proxy Custom Short Form. Customized short forms were scored using this online scoring service: https://www.assessmentcenter.net/ac_scoringservice.

Scoring: Higher scores indicate more fatigue. Each item is scored on a scale of 1–5, and a total fatigue score may range from 10 to 50.

MCID = 2.2 based on 85 parents/guardians of children with cancer, sickle cell disease, nephrotic syndrome or asthma^51^.

#### Parenting stress

##### APSI (paper form)

The APSI was developed based on many interviews of parents of children with autism. We selected it because of findings that many children with MPS III develop autism or autistic symptoms during the course of their disease^38,52^. The APSI Items fall into three categories: the core social disability, difficult-to-manage behaviors and physical issues. The APSI measures how much stress the parents are experiencing related to these three categories. The overall APSI scale score has been validated for parents of children with autism and other developmental disabilities^53^.

Scoring: Higher scores indicate greater parenting stress. Each item is scored on a scale of 0–5, and a total APSI score may range from 0 to 99.

MCID = 0.5 based on 139 neurotypical children aged 2–6 years^53^.

#### Disordered movement

##### Disordered movement 7-d log (paper form)

Duration, severity and type (for example, dystonia, chorea and other) of movement abnormality were reported by the caregiver in real time for 1 week at a time in a survey.

Duration was quantified as:

- Occasional (<25% of the time) = 1
- Intermittent (25–50% of the time) = 2
- Frequent (50–75% of the time) = 3
- Constant (>75% of the time) = 4

Severity was quantified as:

- The movement has interfered less with my child’s daily activities = 1
- No change in the severity of the abnormal movement = 2
- The movement has interfered more with my child’s daily activities = 3

Scoring: Higher scores indicate increased duration and/or severity of disordered movement. Average was taken of the 7-d average Duration score and the 7-d average Severity score. The total score may range from 1 to 7.

MCID = 0.14 based on change in total movement score over the first 8 weeks of observation in this study, before treatment with anakinra (*n* = 23).

#### ICR (paper form)

The ICR allowed a caregiver to choose five out of 15 items that they felt were the most impactful. Items chosen based on previous caregiver preference work^54^ were sleep disturbances, hyperactivity, frustration/impulse control/aggressive behaviors, feeding, anxiety, unhappiness, communication, social deficits, digestive issues and toileting, pain, illness/vulnerability to illness, fatigue, seizure, mobility and gait. The five items selected by the caregiver were then maintained for longitudinal ratings throughout the trial.

The caregiver rated each of these outcomes on a five-point Likert scale as follows:

0 = Not stressful

1 = Sometimes creates stress

2 = Often creates stress

3 = Very stressful on a daily basis

4 = So stressful sometimes we feel we cannot cope

Scoring: Higher scores indicate more stress, with a possible range of 0–20 for the total ICR at each assessment.

### Screening measure

#### Vineland Adaptive Behavior Scales, Second or Third Edition^41,42^ (Second Edition: paper form; Third Edition: paper or electronic form)

The frequency that a person shows independence in age-typical life activities is rated by a caregiver as occurring never, sometimes (or partially) and usually. Domains of Communication, Daily Living Skills and Socialization are combined for an Adaptive Behavior Composite.

Scoring: Norm-referenced scores (population mean ± s.d.) are 100 ± 15. Scores within one standard deviation of the mean—that is, 85–115—are in the ‘average’ range, whereas 70–84 is below average, and less than 70 is impaired. Age-equivalent scores are available from a lookup table in the back of the Vineland manual. The average of all subdomain age equivalents was divided by chronological age to determine if a child was <0.5.

### Post hoc measure

#### Immunophenotyping

Phenotypic characterization of immune cell lineages was performed from whole blood cells (myeloid cells, T cells, T regulatory cells and B regulatory cells) at each study visit to measure immunologic effects of treatment. Whole blood cells were first stained with specific antibodies and then fixed to lyse red blood cells, as previously described^55^. Identification of blood cell subtypes was performed as follows. In brief, monocytes were gated within the myeloid cell population identified by excluding B cells (CD19-APC-Cy7^+^ cells, Thermo Fisher Scientific, 561743) and T cells (CD3-PE^+^ cells, BD Biosciences, BDB555333) from the whole blood population. Within the myeloid population, natural killer (NK) cells were excluded using CD56^+^ cells (CD56-FITC^+^ and CD14^−^ Alexa 647 cells, 340410 and 562690, respectively). Subsequently, neutrophils were identified as bright CD16-PerCP-Cy5.5^+^ (BD Biosciences, 560717) high side scatter (SSC) cells. Monocytes were identified as CD14^+^ and CD16^+^ cells. CD4-APC-Cy7^+^ cells (BD Biosciences, 566319) and CD8-PE-Cy7^+^ cells (BD Biosciences, 335787) were gated within the CD3^+^ cells of the whole blood, excluding B cells (CD19-APC^+^ cells, BD Biosciences, 555415), and B cells were gated as CD19^+^ cells. The antibodies were diluted at a ratio of 1.5:200 and then titrated against blood samples to assess reactivity and binding efficiency. Flow cytometry data were acquired using a BD FACS Aria III using FACSDiva version 9.0.1 and post-analyzed using FlowJo version 10.8.1.

### Statistical analysis

The safety population, used for the safety and tolerability outcomes, included all participants who received at least one dose of anakinra during the study. The efficacy analysis population included all participants assigned to treatment with anakinra regardless of whether they completed the treatment and/or all scheduled study visits (that is, intent-to-treat analysis). This was done to provide the most conservative estimate of treatment effects.

The number of participants for the study was determined by feasibility rather than statistical power owing to the rarity of MPS III. Categorical outcomes were summarized using frequencies and percentages, and continuous outcomes were summarized using means and standard deviations or medians and interquartile ranges.

Mixed models for repeated measures were used to estimate and assess changes over time, with time serving as a within-subject factor. We employed the unstructured covariance structure for the repeated measures and presented the least-squares means along with 95% CIs. Residual analyses were conducted, including studentized and Pearson residuals, and compared to the standard normal quantile–quantile plot to assess the model’s adherence to its underlying assumptions, and no serious violations were detected. Sensitivity analyses were performed using imputation for missing data and a non-parametric test for all six MDRI outcomes. Imputation used the last observation carried forward (LOCF) method (Fig. 3). LOCF is commonly used in clinical trials and longitudinal studies to impute missing values, especially when the missing data do not follow a discernible pattern. Given the limited sample size, identifying a specific pattern of missing data and applying multiple imputation methods was not a viable option due to the lack of data to serve as predictors within a regression model for such imputation. The Wilcoxon signed-rank test was used for non-parametric tests. The association between immunophenotype and the number of improvements and worsenings in MDRI was assessed using Spearman’s rank-based correlation coefficient.

Data were analyzed using SAS version 9.4 (SAS Institute) by author Y.P. All tests were two-tailed, and *P* < 0.05 was considered statistically significant. No adjustment was made for multiplicity in the analyses. Therefore, the reported *P* values and CIs should be interpreted with caution and discussed with recognition of inflated family-wise type I error rate.

### Reporting summary

Further information on research design is available in the Nature Portfolio Reporting Summary linked to this article.

## Online content

Any methods, additional references, Nature Portfolio reporting summaries, source data, extended data, supplementary information, acknowledgements, peer review information; details of author contributions and competing interests; and statements of data and code availability are available at 10.1038/s41591-024-03079-3.

## Supplementary information

Supplementary Information Study Protocol.

Reporting Summary

Supplementary Data 1 Supplementary Data.

## Source data

Source Data Fig. 4 Statistical Source Data.

## Figures and Tables

**Extended Data Table 1:**
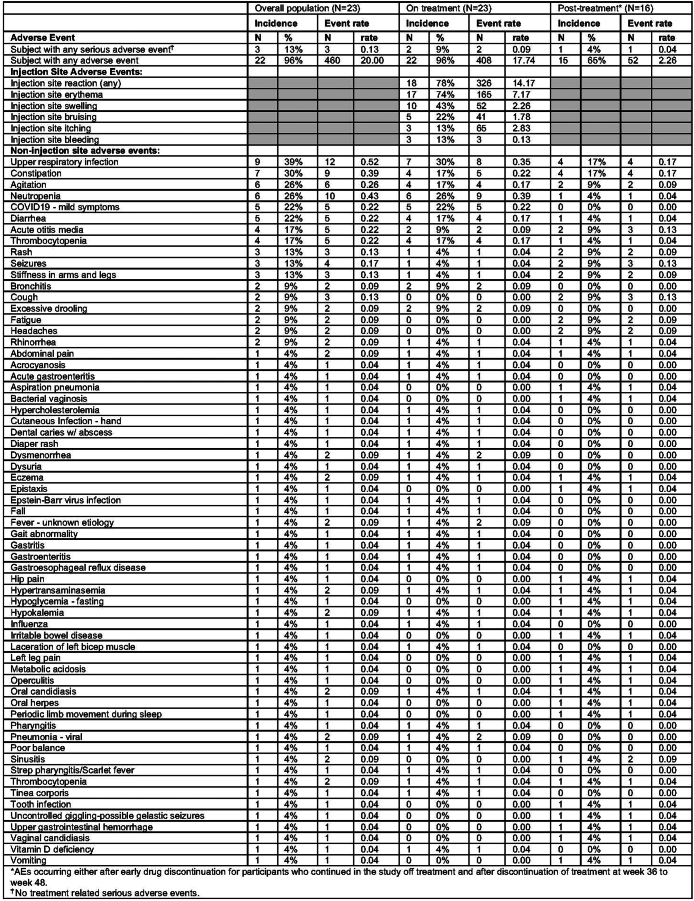
Incidence of and event rates of treatment-emergent AEs by preferred term

**Extended Data Table 2:**
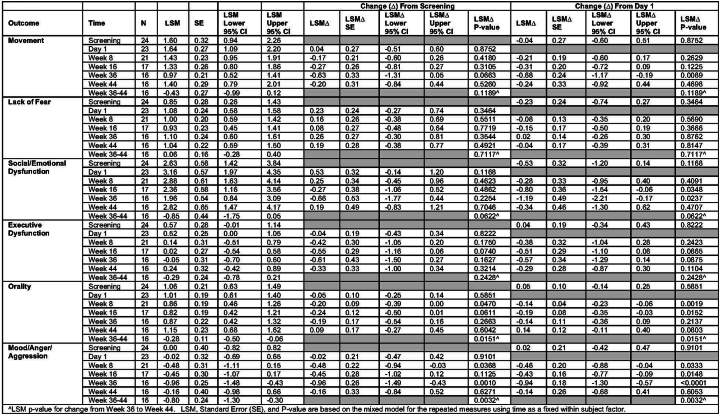
Estimated least-square means (LSMs) in SBRS clusters (Movements, Social/Emotional Dysfunction, Lack of Fear and Executive Dysfunction) and domains (Orality and Mood/Anger/Aggression) before, during and after treatment with anakinra and differences in LSMs from screening and from day 1

All statistical tests were two-sided. No adjustment was made for the multiple comparison.

**Extended Data Table 3:**
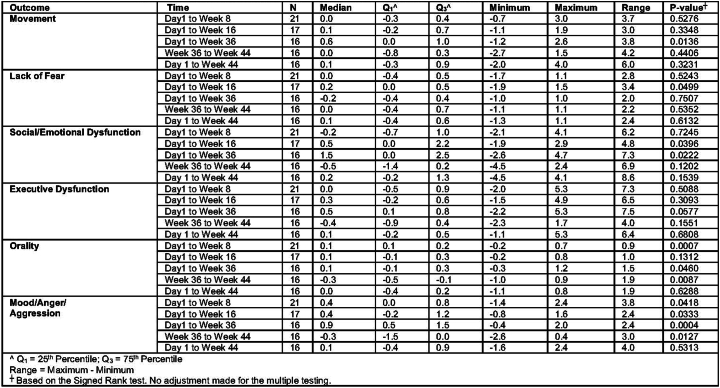
Descriptive statistics of change in SBRS clusters (Movements, Social/Emotional Dysfunction, Lack of Fear and Executive Dysfunction) and domains (Orality and Mood/Anger/Aggression) during and after treatment with anakinra

**Extended Data Table 4:**
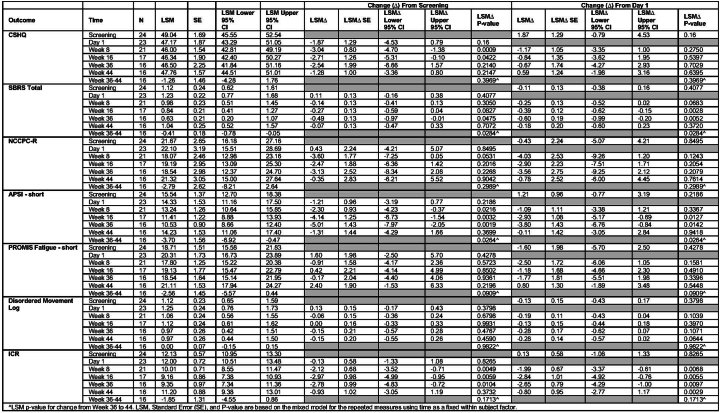
Estimated least-square means (LSMs) in SBRS total score, CSHQ, NCCPC-R, PROMIS Fatigue, APSI, 7-d disordered movement log and the ICR before, during and after treatment with anakinra and differences in LSMs from screening and from day 1

All statistical tests were two-sided. No adjustment was made for the multiple comparison.

**Extended Data Table 5:**
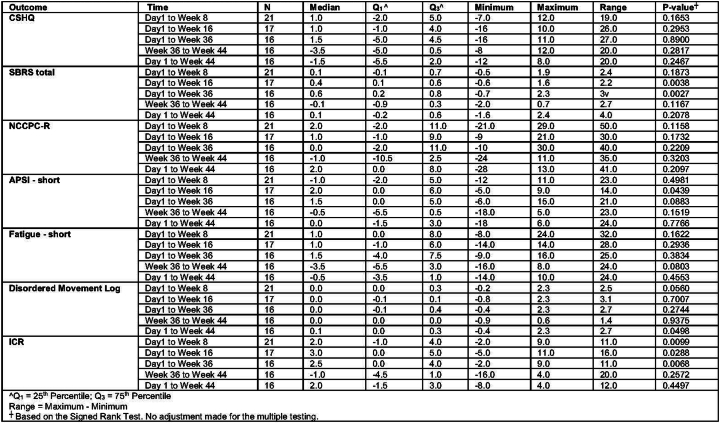
Descriptive statistics of change in six outcomes included in the MDRI (SBRS, CSHQ, NCCPC-R, PROMIS Fatigue, APSI and 7-d disordered movement log) and ICR during and after treatment with anakinra

## Data Availability

The de-identified individual participant data that underlie the results reported in this article (including text, tables and figures) are included in Supplementary Data File 1. Source data are provided with this paper.
