# Author Correction: Anakinra in Sanfilippo syndrome: a phase 1/2 trial

**DOI:** 10.1038/s41591-024-03207-z

**Published:** 2024-07-29

**Authors:** Lynda E. Polgreen, Agnes H. Chen, Youngju Pak, Anna Luzzi, Adolfo Morales Garval, Jonathan Acevedo, Gal Bitan, Michelina Iacovino, Cara O’Neill, Julie B. Eisengart

**Affiliations:** 1 Lundquist Institute for Biomedical Innovation at Harbor-UCLA Medical Center, Torrance, CA USA; 2 Department of Pediatrics, David Geffen School of Medicine, University of California, Los Angeles, Los Angeles, CA USA; 3 Department of Neurology, David Geffen School of Medicine, Brain Research Institute and Molecular Biology Institute University of California, Los Angeles, Los Angeles, CA USA; 4 Cure Sanfilippo Foundation, Columbia, SC USA; 5 Department of Pediatrics, University of Minnesota Medical School, Minneapolis, MN USA

**Keywords:** Phase II trials, Paediatric neurological disorders

Correction to: *Nature Medicine* 10.1038/s41591-024-03079-3, published online 21 June 2024.

In the version of the article initially published, two references were missing and have now been added as refs. 13 and 14: Parker, H. et al. Haematopoietic stem cell gene therapy with IL-1Ra rescues cognitive loss in mucopolysaccharidosis IIIA. *EMBO Mol. Med*. **12**, e11185 (2020) and Mandolfo, O., Parker, H. & Bigger, B. Innate immunity in mucopolysaccharide diseases. *Int. J. Mol. Sci*. **23**, 10.3390/ijms23041999 (2022). Citations of these references have been added to the fourth paragraph of the introduction, and the first paragraph of the Discussion. These corrections have been made to the HTML and PDF versions of the article.

