# Supplementary material for: Anakinra in Sanfilippo syndrome: a phase 1/2 trial
Source: Nat Med. 2024 Jun 21;30(9):2473–9. doi: 10.1038/s41591-024-03079-3 (PMC11405265; doi:10.1038/s41591-024-03079-3)
Supplement: Supplementary file 1 — Study Protocol. [file 41591_2024_3079_MOESM1_ESM.pdf]

nature medicine

Supplementary information https://doi.org/10.1038/s41591-024-03079-3

Anakinra in Sanfilippo syndrome: a phase 1/2 trial

In the format provided by the authors and unedited

PROTOCOL TITLE: *OPEN-LABEL PILOT STUDY OF THE EFFECTS OF ANAKINRA IN MUCOPOLYSACCHARIDOSIS (MPS) III*

SHORT TITLE: Anakinra in MPS III

PRINCIPAL INVESTIGATOR/SPONSOR:
Lynda Polgreen, MD, MS
Lundquist Institute for Biomedical Innovation at Harbor-UCLA Medical Center (LI)
1124 W. Carson St.
Liu Research Bldg.
Torrance, CA 90502

FUNDING SPONSOR: Cure Sanfilippo Foundation

STUDY PRODUCT: anakinra (Kineret™)

IND # 145261

Trial Registration: NCT04018755

IRB# 31834-01

VERSION NUMBER: 1.8
DATE:08-Nov-2023

CONFIDENTIAL
This document is confidential and the property of the LI. No part of it may be transmitted, reproduced, published, or used by other persons without prior written authorization from the study sponsor.

List of Abbreviations

adverse event (AE)
alanine aminotransferase (ALT)
area under the curve (AUC)
aspartate transaminase (AST)
Autism Parenting Stress Index (APSI)
basic metabolic panel (BMP)
body mass index (BMI)
case report form (CRF)
Child Sleep Health Questionnaire (CSHQ)
Clinical and Translational Research Center (CTRC)
Clinical and Translational Science Institute (CTSI)
Common Toxicity Criteria (CTC)
complete blood count with differential (CBCD)
Department of Health and Human Services (DHHS)
ethylenediaminetetraacetic acid (EDTA)
free thyroxine (FT4)
Freedom of Information Act (FOIA)
Food and Drug Administration (FDA)
Genetic Information Nondiscrimination Act (GINA)
Genome Wide Association Studies (GWAS)
glycosaminoglycans (GAG)
Health Insurance Portability and Accountability Act of 1996 (HIPAA)
hematopoietic stem cell transplantation (HSCT)
hurler syndrome (MPS IH)
Individual Clinical Response (ICR)
institutional review board (IRB)
intent to-treat (ITT)
interleukin-1 receptor antagonist (IL-1Ra)
interleukin-1α (IL-1α)
international normalized ratio (INR)
Intravenous immunoglobulin (IVIG)
intrauterine device (IUD)
Investigational Drug Service (IDS)
minimal clinically important difference (MCID)
mucopolysaccharidosis (MPS)
monocyte chemoattractant protein-1 (MCP-1)
multi-domain responder index (MDRI)

National Cancer Institute (NCI)
National Center for Biotechnology Information (NCBI)
National Institutes of Health (NIH)
neonatal onset multisystem inflammatory disease (NOMID)
Non-communicating Children's Pain Checklist-Revised (NCCPC-R)
pharmacokinetic (PK)
Patient Reported Outcomes Measurement Information System (PROMIS)
Protected Health Information (PHI)
Protocol-compliant population (PP)
Rare Disease Clinical Research Group (RDCRG)
Safety population (SP)
Sanfilippo Behavior Rating Scale (SBRS)
Sanfilippo syndrome (MPS III)
serious adverse event (SAE)
serum separator tube (SST)
standard deviation score (SDS)
subcutaneous (SC)
systemic-onset juvenile idiopathic arthritis (SJIA)
thyroid Stimulating Hormone (TSH)
Translational Genomics & Population Sciences (TGPS)
tuberculosis (TB)
unanticipated problems involving risk to subjects or others (UPIRTSO)
upper limit of normal (ULN)
Vineland Adaptive Behavior Scales, Third Edition (VABS-III)

1.0 Version History

Version #	Approval Date	Significant Changes from Previous Version
Version 1.0	10-Oct-2019	Original Protocol Version
Version 1.1		1. Updated compensation plan in section 10.25 to decrease financial burden on study participants and their families.<br>2. Updated institution named from Los Angeles Biomedical Research Institute to The Lundquist Institute.
Version 1.2	12-Mar-2020	3. Added whole blood for immunophenotyping.
Version 1.3	15-May-2020	1. We have changed the Week +8 visit to a telemedicine visit. The rationale for this is that we think given the current COVID-19 pandemic, it is important to limit participant/parent/guardian travel as much as possible. Given the rarity of MPS III and our recruitment from around the country, most of our participants will not live within driving distance of the study center. We do not think this change will increase the risk of the study, as all safety labs and vitals can be done using a home phlebotomy/medical assistant service, the surveys will be sent to the parents by email or mail, and a study physician will perform their evaluations by video conference with the parents/guardian and participant.<br>2. Gait and stair climb assessments have been removed from the Week +8 visit due to the change to a telemedicine visit.<br>3. Grammatical and spelling updates were made.<br>4. Updated Institute name from The Lundquist Institute (TLI) to Lundquist Institute (LI).<br>5. Updated Study Contact email addresses and PI's fax number.<br>6. Added "Screening" visit and "Tanner Stage" procedure under the Schedule of Events.<br>7. Clarification of where videos for Gait Analysis and Stair Climb will be stored.<br>8. Clarification of vital signs per time point.<br>9. Section 5.3: added the following exclusion criteria: Any other social or medical condition that the Investigator believes would pose a significant hazard to the subject if the investigational therapy were initiated or be detrimental to the study.<br>10. Section 10.25: Study subject compensation was clarified.<br>11. Section 10.26: Clarification of preparation and handling and storage of specimens.<br>12. Section 10.2: Updated study design schematic<br>13. Section 10.3: Updated the Schedule of Events
Version 1.4	15-July-2020	1. Section 10.26: Added Lab Corp facility for safety labs.
Version 1.5	04-Sept-2020	1. Section 27.7: Clarified the dose escalation plan. Section 27.7 did not include the dose escalation plan for week

		+16 that is described in the footnote "e" of the Table 1. Schedule of Events. This was added to Section 27.7 to match footnote "e" in Table 1. Schedule of Events and clarify the duration of time until potential treatment discontinuation after escalation to 200 mg SC once daily: "**Dose will be increased to 200 mg SC once daily**, with a maximum dose limit of 8 mg/kg/day, at Week +16 if the change in the two most bothersome outcomes selected by the parents/guardians at the Day +1 visit have worsened by ≥ the MCID as defined in Section 9.1 between week +8 and week +16, after an improvement from Day +1 to week +8. If the MCID is not achieved after 8 weeks on maximum trial dose of 200mg (max dose 8 mg/kg/day), treatment with anakinra will be discontinued."<br>2. We determined that buffy coat is not needed at Weeks +8, +16 and +44 since it is also being collected at Screening, Day +1 and Week +36. Table 1 Schedule of Events was updated to reflect this change.<br>3. Section 10.26: updated procedures for PAXgene tube.<br>4. The visit window for Week 8, 16, 36 and 44 has been changed from ±2 Weeks to ±1 Week.
Version 1.6	09-Dec-2020	1. Sections 10.1 and 10.4: Added Week +24 for safety labs only if the dose is increased at Week +16.<br>2. Section 27.7: Amended protocol to allow for a subject to remain on treatment when there has *not* been an improvement >MCID for the two most bothersome problems chosen by the parents, *if* the effect on one or more study outcomes has improved by >MCID. This will be at the discretion of the parent(s) and principal investigator after they review and discuss all subject specific results.
Version 1.7	11-Jan-2021	1. Administrative changes to section 27.7 to further clarify dosing plan.<br>2. The definition of neutropenia has been changed to <1200 cells/microliter.<br>*Rationale:* Normal reference range from LabCorp is <1200 cells/microliter. ANC levels ≥1200 do not increase participant risk.<br>3. Section 10.13: to improve characterization of movement disorder, added "Optional: The parents can take a short, representative video of the movement disorder on the days they are recording it as above."<br>*Rationale:* Received feedback from parents that the current movement log is not adequately capturing the movement disorder outcome.

		4. Section 9.1: Removed seizures from the MDRI and list of choices for 2 most bothersome problems. Also changed measurement of seizure outcome to continuous real-time recording by caregivers.<br>*Rationale:* We have found seizures are uncommon in our cohort and if present, are well controlled on anti-epileptics and/or so sporadic that the 1-week logs are not adequately capturing this outcome. Given the rarity of these, we believe capturing each event during the entire study will provide a better evaluation of this outcome.
Version 1.8	08-Nov-2023	1. Administrative changes to Section 3.0 and 9.1 to further clarify that the MDRI is part of the method for determining need for dose escalation (i.e., Phase 2 primary outcome).<br>- Section 3.0 Added wording to elaborate that primary objective #2 is "a means to determine need for dose escalation." Added further elaboration that this is because of the clinical heterogeneity of the population: "Due to the inherent heterogeneity of disease presentation, the MDRI will measure change tailored to patient-specific symptoms. MDRI change will inform dose-finding by reflecting lack of change in clinical outcome. The MDRI will be composed of the following:"<br>*Rationale:* Received feedback that wording is unclear as to relationship of MDRI to dose finding.<br>- Section 9.1 Added wording to clarify that the Primary Endpoint for the Phase 2 is dose finding: "Need for dose escalation as determined by within individual change over 8-week treatment period compared to change over 8-week observational period in the 2 most bothersome symptoms for each enrolled patient, selected from measures for the MDRI."<br>*Rationale:* Received feedback that wording is unclear as to relationship of MDRI to dose finding.<br>2. Administrative changes in Section 2.2 to reflect change in title from Assistant Professor to Associate Professor for Dr. Julie Eisengart.

2.0 Study Contact Information

2.1 PRINCIPAL INVESTIGATOR (PI) / SPONSOR CONTACT INFORMATION

Lynda Polgreen, MD, MS
Investigator/Associate Professor of Pediatrics
Pediatric Endocrinology
LI
1124 West Carson Street
Liu Research Bldg.
Torrance, CA 90502
Telephone: 310-222-1961; Fax: 310-972-2962
lpolgreen@lundquist.org

2.2 CO-INVESTIGATORS CONTACT INFORMATION

Agnes Chen, MD
Investigator/Associate Professor of Pediatrics
Chief, Division of Pediatric Neurology
LI
1124 West Carson Street
N-25
Torrance, CA 90502
Telephone: 310-222-4160
ahchen@lundquist.org

Julie Eisengart, PhD, LP
Investigator/Associate Professor of Pediatrics
Pediatric Neuropsychology
Division of Clinical Behavioral Neuroscience
717 Delaware St., SE, Suite 353
Minneapolis, MN 55414
Telephone: 612-625-6186
eisen139@umn.edu

Cara O'Neill, MD
Chief Science Officer
Cure Sanfilippo Foundation
PO Box 6901
Columbia, SC 29260
Telephone: 803-528-1150

Cara.curesff@gmail.com

2.3 KEY STUDY PERSONNEL

Clinical Coordinators:
Eva Villa-Lopez
Adolfo Morales
LI
1124 W. Carson St., RB3
Torrance, CA 90502
Telephone: 310-803-5650
Fax: 310-972-2962
evilla@lundquist.org
amorales@lundquist.org

Regulatory Specialist:
Angel "Jun" Zozobrado
LI
1124 W. Carson St., RB3
Torrance, CA 90502
Telephone: 310-781-3652
Fax: 310-972-2962
azozobrado@lundquist.org

Program Coordinator:
Cristi Williams
LI
1124 W. Carson St., E5
Torrance, CA 90502
Telephone: 310-781-1407
Fax: 310-972-2962
cristi.williams@lundquist.org

Biostatistician:
Youngju Pak, Ph.D.
Associate Professor of Biostatistics
UCLA Clinical and Translational
Science Institute
LI
1124 West Carson Street, RB-1
Torrance, CA 90502
Telephone: 310-222-1874
ypak@lunquist.org

Table of Contents

1.0 Version History **4**

2.0 Study Contact Information **7**

- 2.1 PRINCIPAL INVESTIGATOR (PI) / SPONSOR CONTACT INFORMATION 7
- 2.2 CO-INVESTIGATORS CONTACT INFORMATION 7
- 2.3 KEY STUDY PERSONNEL 8

3.0 Objectives **12**

4.0 Background **13**

5.0 Inclusion and Exclusion Criteria **14**

- 5.1 How participants will be screened 14
- 5.2 Inclusion criteria 15
- 5.3 Exclusion 16
- 5.4 Special populations 17

6.0 Study-Wide Number of Subjects **17**

7.0 Study-Wide Recruitment Methods **17**

8.0 Study Timelines .... **18~~17~~**

9.0 Study Endpoints .... **18~~17~~**

- 9.1 Primary and secondary endpoints. 18

10.0 Procedures Involved .... **21~~20~~**

- 10.1 Study design .... 21~~20~~
- 10.2 Study Timeline/Schedule of Events 22
- 10.3 Medical History 23
- 10.4 Laboratory Evaluations 23
- 10.5 Physical examination and Anthropometrics 24
- 10.6 Sleep 24
- 10.7 Stooling 25
- 10.8 Sanfilippo Behavior Rating Scale (SBRS) 25
- 10.9 Non-communicating Children's Pain Checklist-Revised (NCCPC-R) 25
- 10.10 NIH Patient Reported Outcomes Measurement Information System (PROMIS) 25
- 10.11 Autism Parenting Stress Index (APSI) .... 26~~25~~
- 10.12 Seizures 26
- 10.13 Disordered Movement 26
- 10.14 Individual Clinical Response (ICR) 26
- 10.15 Gait Analysis .... 27~~26~~
- 10.16 Vineland Adaptive Behavior Scales, Third Edition (VABS-III) 27
- 10.17 Stair Climb 27
- 10.18 Stool Microbiome 27
- 10.19 Lumbar puncture and CSF collection .... 28~~27~~
- 10.20 Unscheduled Study Visits 28
- 10.21 Safety Procedures .... 29~~28~~
- 10.22 Drugs used in the research and the purpose of their use, and their regulatory approval status. .... 30~~29~~
- 10.23 The source records that will be used to collect data about subjects 30
- 10.24 What data will be collected including long-term follow-up. 30
- 10.25 Subject Compensation 30
- 10.26 Laboratory Testing Procedures 30

11.0 Data and Specimen Banking **32**

12.0 Data Management and Confidentiality 32
12.1 Subject Population(s) for Analysis 32
12.2 Analysis .... 33~~32~~
12.3 Power .... 34~~33~~
12.4 Database 34
12.5 Data Quality Control 34

13.0 Provisions to Monitor the Data to Ensure the Safety of Subjects .... 35~~34~~
13.1 Additional Provisions to Monitor Genetic Data 35
13.2 Definitions for recording/reporting adverse events 36
13.3 Recording of Adverse Events 38
13.4 Study Stopping Rules .... 39~~38~~
13.5 Reporting of Serious Adverse Events 39

14.0 Withdrawal of Subjects .... 41~~40~~

15.0 Risks to Subjects .... 43~~42~~
15.1 Venipuncture .... 43~~42~~
15.2 Anakinra .... 43~~42~~

16.0 Potential Benefits to Subjects 43

17.0 Sharing of Results with Subjects 43

18.0 Setting .... 44~~43~~

19.0 Resources Available .... 44~~43~~

20.0 Prior Approvals .... 45~~44~~

21.0 Recruitment Methods .... 45~~44~~

22.0 Local Number of Subjects .... 45~~44~~

23.0 Provisions to Protect the Privacy Interests of Subjects .... 45~~44~~

24.0 Compensation for Research-Related Injury .... 46~~45~~

25.0 Economic Burden to Subjects .... 46~~45~~

26.0 Consent Process .... 46~~45~~
26.1 Subjects who are not yet adults .... 46~~45~~
26.2 Cognitively Impaired Adults /Adults Unable to Consent .... 47~~46~~
26.3 The process for assent of the subjects .... 47~~46~~
26.4 Process to Document Consent in Writing 47
26.5 Vulnerable Populations .... 48~~47~~

27.0 Specific Drug Supply Requirements .... 48~~47~~
27.1 Drug Receipt .... 48~~47~~
27.2 Packaging .... 48~~47~~
27.3 Storage .... 48~~47~~
27.4 Dispensing of Study Drug .... 48~~47~~
27.5 Parent training .... 49~~48~~
27.6 Return or Destruction of Study Drug .... 49~~48~~
27.7 Dosing .... 49~~48~~
27.8 Investigational New Drug Application .... 51~~50~~
27.9 Publication plan .... 51~~50~~

28.0 References .... 53~~52~~
APPENDIX A: Data and Safety Monitoring Plan (Single-Centered, No DSMB) .... 57~~56~~

3.0 Objectives

The primary objectives of the study are to:

1. Evaluate the safety and tolerability of anakinra in children age ≥4 years with MPS III, defined by the incidence of treatment-emergent adverse events and abnormal laboratory values, and number of subjects withdrawn due to intolerability (e.g. unable to administer SC injection to subject, worsening behaviors).

2. Evaluate the effect of anakinra on a multi-domain responder index (MDRI) as a means to determine need for dose escalation. Due to the inherent heterogeneity of disease presentation, the MDRI will measure change tailored to patient-specific symptoms. MDRI change will inform dose-finding by reflecting lack of change in clinical outcome. The MDRI will be composed of the following:
   - Sanfilippo Behavior Rating Scale (SBRS)
   - Child Sleep Health Questionnaire (CSHQ)
   - Autism Parenting Stress Index (APSI)
   - PROMIS Fatigue - Parent Proxy Custom Short Form
   - Seizures – parent reported frequency, duration, and severity
   - Movement disorder (e.g. dystonia, chorea, etc.) – parent reported frequency, duration, and severity
   - Non-communicating Children's Pain Checklist-Revised (NCCPC-R)

The secondary objectives of the study are to evaluate the effect of anakinra on:

- Individual Clinical Response (ICR) – 5 most impactful clinical problems reported by the caregiver
- Gait
- Stair climb/descent
- Sleep Diary (1-week recall)
- Sanfilippo Syndrome Stool Habit Questionnaire

The exploratory objectives of the study are to evaluate the effect of anakinra on:

- Stool microbiome
- Vineland Adaptive Behavior Scales, Third Edition (VABS-III)

4.0 Background

Sanfilippo syndrome, or mucopolysaccharidosis type III (MPS III), is a genetic disorder of metabolism, associated with insufficient production of a lysosomal enzyme crucial for degradation of heparan sulfate. As a consequence, buildup of heparan sulfate causes progressive, irreversible neurodegeneration. There are four variants of MPS III, distinguished by the lysosomal enzyme deficiency: Heparan-N-sulfatase (MPS IIIA), a-N-Acetylglucosaminidase (MPS IIIB), AcetylCoA:N-acetyltransferase (MPS IIIC) and N-Acetylglucosamine 6-sulfatase (MPS IIID). MPS IIIA and MPS IIIB are the most common subtypes.

Unlike other types of MPS which involve considerable somatic complications, the most significant aspect of MPS III is neurodegeneration which manifests in stages of developmental disruption and decline, defined by Cleary and Wraith[1]. Their 3-stage model involves: 1) developmental delay in cognition and language before age 3-4 years, 2) temper tantrums, hyperactivity, aggression, sleep disorder, and a further loss of cognitive and language function from age 3-4 years to age 8-10 years, and 3) loss of motor function, feeding difficulties, and seizures from age 8-10 years. Recent work has determined that there is a loss of about 15 IQ points per year in children with MPS IIIA who are younger than 6 years[2] and that they do not surpass a mental age equivalent of 30 months. The advancing disease causes the neurobehavioral profile to meet diagnostic criteria for autism[3], and to overlap with Kluver-Bucy syndrome[4]. A study of patients with both MPS IIIA and IIIB in several European countries found comparable patterns of disease progression in the groups, with delayed language development and cognitive decline, as well as disruptive behavior, particularly in children diagnosed before age 5[5].

The neurobehavioral symptoms associated with MPS III are highly disruptive and distressing to families, have a significant impact on the quality of life for the patient and family[6], and likely interfere with adjunctive therapeutic attempts at supporting the child. However, despite the universal acknowledgment that these neurobehavioral symptoms are a prominent concern for MPS III, these symptoms have not been the therapeutic target.

Sanfilippo syndrome is the most common form of MPS, with an incidence of about 1 in 70,000 births. However, to date no evidence-based treatments are available, which is a striking contrast to another neurodegenerative form of MPS, Hurler syndrome (MPS IH),

which is much rarer but can be arrested with hematopoietic stem cell transplantation (HSCT). HSCT was tried for MPS III, but all patients failed to obtain any benefit[7]. A trial of Genistein, an over-the-counter supplement that apparently can lower glycosaminoglycans, such as heparan sulfate, in the urine, was recently closed due to

lack of clinical benefits. In addition, an ongoing study of caregiver preference focused on meaningful treatment benefit outcomes is underway. Focus group data indicated that caregivers prioritized symptoms of communication problems, difficult behaviors, pain, declining mobility, poor sleep and gastrointestinal issues as desired targets of potential emerging therapies[8]. While new therapies are proliferating, there are many affected children who are ineligible for clinical trials but whose families are suffering as the disease advances and the neurobehavioral profile becomes profoundly disruptive.

Inflammation and Sanfilippo Syndrome

Neurologic and somatic inflammation have been implicated in disease pathogenesis in the MPS disorders [9–13]. For Sanfilippo syndrome, elevation in levels of interleukin-1α (IL-1α) and monocyte chemoattractant protein-1 (MCP-1) have been reported in the MPS IIIB mouse model in both brain and plasma[14]. Despite less visually obvious somatic sequelae compared to other forms of MPS, patients with Sanfilippo syndrome experienced the highest frequency of pain in a Dutch national survey as measured by the Non-communicating Children's Pain Checklist-Revised (NCCPC-R)[15]. It is widely understood that inflammation is a source of pain and due to the known inflammatory nature of accumulated GAG fragments, addressing inflammation is a rational approach to improving quality of life. Thus, adjunctive therapies aimed at decreasing inflammation are currently being studied in many MPS disorders and benefits in both somatic and CNS disease have been reported[9,12,14,16]. For example, treatment with prednisolone alone has been shown to correct behaviors in MPS IIIB mice[14], and inhibition of IL-1 through gene therapy-induced overexpression of the human IL-1 receptor antagonist (IL-1Ra) results in a reduction in the number of activated microglia and astrocytosis, and significant improvements in hyperactivity and working memory in MPS IIIA mice[12]. These studies provide the rationale for testing the effects of anakinra, an IL-1 receptor antagonist, in children with MPS III.

We hypothesize that anakinra will reduce hyperactivity and irritability, as well as improve sleep, in children with MPS III.

5.0 Inclusion and Exclusion Criteria

5.1 How participants will be screened

Potential participants will be pre-screened by phone or email with the following questions:

1. *Does your child have sleep problems that make him or her get less sleep than needed? Y/N*
2. *Does your child have challenging behaviors that make it difficult to go on outings or manage the child's daily needs? Y/N*
3. *Does your child have pain or unexplained episodes of distress? Y/N*
4. *Does your child have seizures or movement disorders? Y/N*
5. *Does your child have more difficulty than a same-aged healthy child walking and getting around independently? Y/N*
6. *Does your child require more supervision than a same-aged healthy child to keep them safe? Y/N*

If in the opinion of the investigator, the patient may be a candidate for an enzyme restorative trial that is enrolling participants, the caregiver will be directed to information on that trial(s) at clinicaltrials.gov. This will also be assessed at the screening visit using the inclusion criteria described below which are in part based on exclusion criteria from currently active enzyme restorative trials.

If the answer to 2 or more of these is "yes", then we will schedule a screening visit at our center. Disease burden in these areas will be confirmed through the protocol specified outcome measures.

At the Screening visit consent and assent forms will be reviewed in detail and the patient and their parents/legal guardians will have the opportunity to ask any questions they may have. The parents will also be asked to describe the protocol prior to signing these forms to ensure that all details are clearly understood. Screening evaluations will then be performed as detailed in Table 1.

5.2 Inclusion criteria

- MPS III diagnosis confirmed by genetic testing.
- ≥ 4 years of age.
- Patient or parent/legal guardian is able and willing to provide informed consent. For patients 7 to 17 years of age, assent must also be provided when cognitively possible.
- If on Genistein, must have been on a stable dose for 6 months prior to enrollment.
- If on melatonin or other sleep medications, must have been on stable doses for the past 3 months.

- Two of the following criteria are met:
  1. CSHQ Total score ≥ 41[17,18].
  2. SBRS Cluster or Domain score ≥ -2 SD of mean for age group[19].
  3. The presence of significant MPS III related CNS impairment or behavioral disturbances.
  4. NCCPC-R Total Score of ≥ 7[20].
  5. Seizure disorder thought to be due to MPS III related disease changes, requiring use of regular medication.
  6. Presence of a movement disorder.

  OR, one of the following criteria are met:
  1. Previous participation in a gene/cell therapy or enzyme restorative clinical trial.
  2. Previous rejection from a gene/cell therapy or enzyme restorative clinical trial.
  3. Functional age as measured by the Vineland is ≤ 0.5 chronological age.

5.3 Exclusion

- Currently enrolled in another ongoing clinical treatment trial.
- Previous or current treatment with anakinra, canakinumab or any other IL-1 inhibitor.
- Use of the following therapies prior to enrollment:
  - Narcotic analgesics within 24 hours prior to enrollment.
  - Tocilizumab, dapsone or mycophenolate mofetil within 3 weeks prior to enrollment.
  - Etanercept, leflunomide, thalidomide, or cyclosporine or intraarticular, intramuscular, intravenous, or oral administration of glucocorticoids within 4 weeks prior to enrollment.
  - Intravenous immunoglobulin (IVIG), adalimumab, or methotrexate within 8 weeks prior to enrollment.
  - Infliximab, 6-mercaptopurine, azathioprine, cyclophosphamide or chlorambucil within 12 weeks prior to enrollment.
  - Rituximab within 26 weeks prior to enrollment.
- Live vaccines within 1 month prior to enrollment.
- Known presence or suspicion of active, chronic or recurrent serious bacterial, fungal or viral infections, including tuberculosis, HIV infection or hepatitis B or C infection.
- Clinical evidence of liver disease or liver injury as indicated by presence of abnormal liver tests.

- AST or ALT > 5 x ULN, or
- AST or ALT > 3 x ULN accompanied by elevated bilirubin >2 x ULN.

- Presence of severe renal function impairment (estimated creatinine clearance < 30 mL/min/1.73m2).
- Presence of neutropenia (defined as ANC <1200 cells/microliter).
- History of malignancy.

- Known hypersensitivity to *E. coli*-derived proteins, or any components of Kineret™ (anakinra).
- Pregnant or lactating women.
- Current active infection.
- History of serious opportunistic infection (e.g. bacterial [Legionella and Listeria]; tuberculosis [TB]; invasive fungal infections; or viral, parasitic, and other opportunistic infections).
- Positive TB skin test, positive Quantiferon-TB Gold TB test, positive chest x-ray, or a recent exposure to TB.
- Requirement for live vaccine exposure that would be expected to occur during the time frame of the study.
- Any other social or medical condition that the Investigator believes would pose a significant hazard to the subject if the investigational therapy were initiated or be detrimental to the study.

5.4 Special populations

- Adults unable to consent will be included.
- Individuals who are not yet adults (infants, children, teenagers) will be included.
- Pregnant women will not be included.
- Prisoners will not be included.

6.0 Study-Wide Number of Subjects

Twenty (20) subjects will be enrolled in this pilot study. Individuals who withdraw before Week +8 will be replaced.

7.0 Study-Wide Recruitment Methods

Potential subjects will be recruited through study physicians' and other MPS physicians' clinical practices. The study will be registered on clinicaltrials.gov. Information on the study will be shared through social media as well.

8.0 Study Timelines

- The duration of an individual subject's participation in the study will be 52 weeks.
- The duration anticipated to enroll all study subjects is 1 year.
- The estimated date for the investigators to complete this study (complete primary analyses) is 3 months after the last subject's Week +8 visit.

9.0 Study Endpoints

9.1 Primary and secondary endpoints.

Primary endpoints:

Phase 1: Incidence of treatment-emergent adverse events and laboratory values over 8 weeks of treatment to known frequency of anakinra-related AEs in other populations.

Phase 2: Need for dose escalation as determined by within individual change over 8-week treatment period compared to change over 8-week observational period in the 2 most bothersome symptoms for each enrolled patient, selected from measures for the MDRI. Choices are below in bold. These 2 symptoms will be monitored by one or more of the assessments below. If the Minimal Clinically Important Difference (MCID) that is defined below is not achieved after 8 weeks of treatment, or there has been a decrease of more than the MCID between 8 and 16 weeks after an increase greater than MCID at week 8, the dose will be escalated (see Section 27.7). If the MCID is not achieved after 8 weeks on maximum trial dose of 200mg (max dose 8 mg/kg/day), treatment with anakinra may be discontinued (see Section 27.7).

- Sanfilippo **Behavior** Rating Scale (SBRS): MCID = 0.25 SD for each scale[19]
    - Symptoms described in table below:

Body Movements	Makes large complex movements of their body, such as spinning, twirling or repeatedly bouncing up and down.	Movement
Object Interaction	Examines or manipulates some novel or unfamiliar objects repeatedly.	
Activity/Routines	Never plays with one object or engages in one activity for very long.	
Emotional Function	Expresses emotions inappropriate to the situation.	Social/emotional function
Eye Contact	Makes effort to avoid other people's gaze (keeps head down, looks away, or turns away).	
Social Interaction	Participates in group activities.	
Emotional Engagement	Doesn't seem to recognize or respond to other people's emotion.	
Comfort Seeking	Actively seeks comfort from familiar people.	
Safety Consciousness	Is less aware of danger around the house (e.g. would touch hot oven) on the street or in the playground than other children their age.	Lack of Fear
Fearfulness	Is less fearful of new strange or scary things than other children of same age.	
Attention	Is easily distracted	Executive function
Self-Control/Compliance	Is impulsive, can't stop self.	
Orality	Puts everything in their mouth; eats inedible things.	
Mood/Anger/Aggression	Mood is irritable and cranky.	
Self-Gratification	Rubs genitals with hands, inside or outside clothing	

- Child **Sleep** Health Questionnaire (CSHQ): MCID = 1-point change on the CSHQ Total score [17]
- Autism **Parenting Stress** Index (APSI): MCID = 0.25 SD for Stress Total [21]
    - Ability to communicate
    - Tantrums/meltdowns
    - Self-injurious behavior
    - Difficulty making transitions from one activity to another
    - Toilet training
    - Not feeling close to your child
    - Concern for your child not being accepted by others
    - Concern for the future for your child living independently
- PROMIS **Fatigue** - Parent Proxy Custom Short Form: MCID = 3-point change
- **Seizures** – Parents will record each seizure in a seizure journal provided by the study. Information recorded will include the duration (average length of seizure), and need for medical intervention (e.g. rescue medication, medical evaluation).

- **Movement** disorder (e.g. dystonia, chorea, etc.) – Parent reported duration and severity. Change in one of two characteristics for +/-1 score, +/-2 score for change in two of the characteristics.
- Non-communicating Children's **Pain** Checklist-Revised (NCCPC-R): MCID = 0.25 SD[20]

Secondary Endpoints:

Within individual change over 8-week treatment period (on maximum effective and tolerated dose) compared to change over 8-week observational period, and from Day 0 to Week +36 (i.e. treatment phase) in:

1. Incidence of treatment-emergent adverse events and laboratory values over 36-weeks of treatment to known frequency of anakinra-related AEs in other populations.
2. MDRI comprised of:

- Sanfilippo Behavior Rating Scale (SBRS): Minimal Clinically Important Difference (MCID) = 0.25 SD for each scale[19]
- Child Sleep Health Questionnaire (CSHQ): MCID = 1-point change [17]
- Autism Parenting Stress Index (APSI): MCID = 0.25 SD for Stress Total [21]
- PROMIS Fatigue - Parent Proxy Custom Short Form: MCID = 3-point change
- Movement disorder (e.g. dystonia, chorea, etc.): Parent reported duration and severity. Change in one of two characteristics for +/-1 score, +/-2 score for change in two of the characteristics.
- Non-communicating Children's Pain Checklist-Revised (NCCPC-R): MCID = 0.25 SD[20]

The MDRI will be calculated as the sum of scores from the components. Subjects will receive a score for each component of the MDRI listed above. A score of 0 will be given for a change in score less than the MCID. A score of +1 (improvement) or -1 (worsening) will be given for a change in score of ≥ 1 MCID and < 2 times MCID. A score of +2 (improvement) or -2 (worsening) will be given for a change in score of ≥ 2 and <3 times MCID. A score of +3 (improvement) or -3 (worsening) will be given for a change in score of ≥ 3 times MCID. Seizures and movement disorder outcomes will be scored as described above.

3. Individual Clinical Response (ICR) – 5 most impactful clinical problems reported by the caregiver. Sum using 5-point Likert scale for each ICR element.

4. Gait: Qualitative assessment by physical therapist (i.e. improved, no change, worsened).
5. Stair climb/descent: Time and quality of movement.
6. Sleep Diary (1-week recall): Sum of the number of overnight awakenings, average duration of overnight awakenings, and duration of time for initial sleep onset at bedtime.
7. Stooling Survey.

Exploratory Endpoints:

Within individual change from Day 0 to Week +36 (i.e. treatment phase) in:

- Stool microbiome
- Adaptive behavior function: Vineland Adaptive Behavior Scales, Third Edition (VABS-III) – Day 0 to Week 3

10.0 Procedures Involved

10.1 Study design

This is an open label, single center, pilot study of 20 participants with MPS. There will be an initial screening visit, followed by an 8-week observational period, then a

36-week treatment period, and finally another 8-week observational period to determine any effects of withdrawal from the treatment. Total duration of subject participation is 52 weeks.

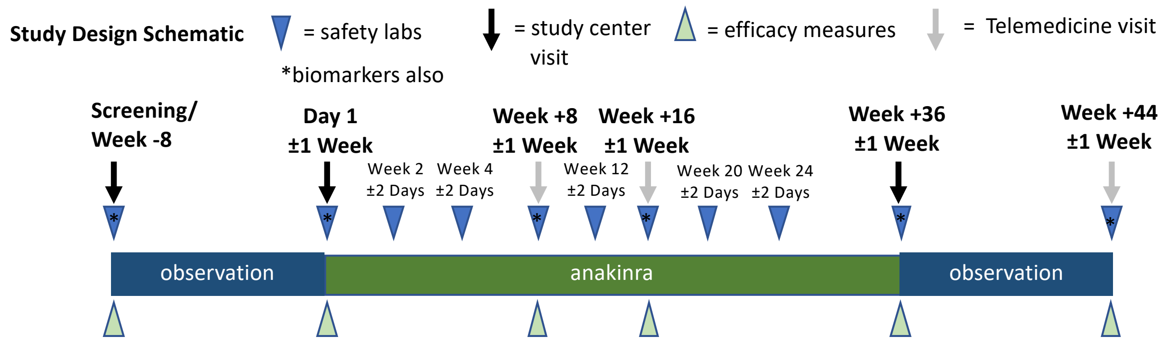

10.2 Study Timeline/Schedule of Events

Procedure	Screening	Week -8	Day +1 ±1 week	Weeks +2, +4, +12, +20 and +24[k] ±2 days	Week +36[a] ±1 week	Weeks +8, +16 and +44 ±1 week
Informed consent	X					
Medical history[b]/Physical exam	X	X	X		X	X
Pubertal assessment (Tanner staging)		X			X	
Concomitant medications[c]	X	X	X		X	X
Pregnancy test	X	X	X		X	X
Vital signs, height, weight	X	X	X		X	X
Child Sleep Health Questionnaire		X	X		X	X
Sanfilippo Behavior Rating Scale		X	X		X	X
Non-communicating Children's Pain Checklist-Revised		X	X		X	X
Parent reported outcomes (PROMIS)[d]		X	X		X	X
Seizure - parent assessment x 1 week		X[h]	X		X	X
Movement - parent assessment x 1 week		X[h]	X		X	X
Individual Clinical Response		X	X		X	X
Gait assessment		X	X		X	
Vineland-III		X			X	
Stair climb		X	X		X	
Sleep Diary – parent assessment x 1 week		X[h]	X		X	X
Stooling Survey		X	X		X	X
Safety labs (CBCD, BMP, LFT, lipid panel)[i]	X	X	X	X	X	X
Thyroid studies	X					
Blood biomarkers		X	X		X	X
Urine biomarkers		X	X		X	X
Stool collection		X	X		X	X
Genetic biomarkers (optional)		X	X		X	X[j]
CSF collection (optional)		X	X		X	X (week 44 only)
Whole Blood		X	X		X	X
Anakinra[e]						X (week 8 and 16 only)
Adverse events[f]		X	X		X	X
Contact for study follow up[g]		Required every 4 weeks ± 7 days				

[a] If sponsor discontinues the study at any time, or a subject is discontinued from the study after week +8 and before week +44, an early termination visit will be done. Early termination visit procedures will be the same as for the week +44 visit.
[b] All procedures/intervention/surgery due to underlying MPS III will be recorded after informed consent is obtained, until week 44 or the Early Termination visit. If a subject is discontinued from the study prematurely, all procedures/intervention/surgery due to underlying MPS III will be recorded at the Early Termination visit.
[c] All medications (prescription, over-the-counter and herbal) and nutritional supplements taken 30 days prior to Screening and throughout the study should be documented.
[d] PROMIS surveys are the Autism Parenting Stress Index (APSI) and a customized Parent Proxy Fatigue – Short Form
[e] The same injection site should not be used 2 days in a row, and should be rotated between the four injection sites (upper thigh, upper back of arm, abdomen or buttocks). Dose will be escalated at week 8 and 16, or decreased at any time, pre protocol described in Section 27.7 Dosing.
[f] After written informed consent but before study treatment initiation, only SAEs associated with protocol-imposed interventions will be recorded. After study drug initiation, all AEs, SAEs, and pregnancy will be recorded until wee 44 or the Early Termination visit. If a subject is discontinued from the study prematurely, AEs and SAEs will be recorded at the Early Termination visit.
[g] During these contacts, study staff will ask about dose administration and seek information on AEs and SAEs by specific questioning. Information on all AEs and SAEs should be recorded in the subject's medical record and on the AE eCRF.
[h] Diaries will be completed at week -6 to avoid the potential impact of travel on these outcomes.
[i] Plasma and serum samples collected and stored for PK and anti-drug antibody studies.
[j] Buffy coat will only be collected on Week -8, Day 1, and Week +36.
K Week +24 safety labs only if dose was increased at week +16.

10.3 Medical History

A study physician and/or study coordinator will review the following with the subject/parent or legal guardian:

- Current and past prescription medications and doses
- Current and past medical history
- Review of systems
- Family history

The subject's medical history will be reviewed at each study visit. This will include query of the subject and parent or legal guardian (as applicable) regarding any changes since the last study visit. If a participant has a greater than 2-week history of cough a CXR will be obtained to screen for active TB.

10.4 Laboratory Evaluations

Blood and urine will be collected at the time points described in the Schedule of Events.

Complete blood count with differential (CBCD), liver function tests (LFTs), lipid panel, and basic metabolic panel (BMP) will be performed at Quest Diagnostics or LabCorp at Screening, Week -8, Day 1, Week +2, Week +4, Week +8, Week +12, Week +16, Week +20, Week +24 (only if dose increased at week +16), Week +36, and Week +44. Thyroid function tests (TSH, Free T4) and Quantiferon-TB Gold test will be obtained at Screening.

Anti-anakinra antibodies will not be measured during this pilot study; the rationale for this is based on prior studies showing no effect of anti-anakinra antibodies on pharmacokinetic (PK) parameters, efficacy outcomes or adverse events (AEs)[22] and low incidence of neutralizing anti-anakinra antibodies[23]. However, serum will be collected at Day 1, Week +8, Week +36, and Week +44 and stored for possible future testing of anti-anakinra antibodies if needed for interpretation of safety or efficacy results.

Plasma will be collected and stored for future PK studies at Day 1, Week +8, Week +36, and Week +44.

For post-menarche female patients, a urine pregnancy test will be performed at Screening and at each study visit.

Plasma, serum, urine, and PAXgene tube for RNA will be collected for future biomarker studies at Week -8, Day 1, Week +8, Week +16, Week +36, and Week +44. Consent will be requested to perform these additional biomarker studies on stored samples in the future as well as genome wide association studies (GWAS) and/or large-scale genomics/transcriptomics assays such as sequencing, methylation and expression.

Buffy coat for DNA will be collected for future biomarker studies at Week -8, Day 1, and Week +36.

Stool will be collected at Week -8, Day 1, Week +8, Week +16, Week +36, and Week +44 for future microbiome studies.

Whole blood will be collected at Week -8, Day 1, Week +8, Week +16, Week +36 and Week +44.

10.5 Physical examination and Anthropometrics

A trained pediatric provider will perform a physical examination at each visit that will include a detailed lymph node assessment. Tanner staging of pubertal development, including breast and pubic hair development in girls, and testicular size and pubic hair development in boys will be performed each study visit.

Standard anthropometric measurements including standing height and weight will be performed. Height or supine length if unable to stand will be measured 3 times standing without shoes. The average of 3 measurements by the same observer using identical technique with a wall-mounted stadiometer will be recorded. Each subject will be repositioned between each measurement. Height standard deviation score (SDS) will be calculated based on the National Center for Health Statistics 2000 data as provided by the Center for Disease Control. Weight to be measured standing, once with subject in light clothing or dressing gown with shoes removed. Height and weight measurements will be used to calculate body mass index (BMI) as weight (kg) divided by height (m^2).

10.6 Sleep

Sleep will be assessed using two methods:

1. <u>Sleep Diary</u> – Parents will record daily for 1 week during Week -6 (to avoid the effects of travel on sleep) and then 1 week prior to each study visit after that, the following information:
   - Number of night time awakenings
   - Duration of nighttime awakenings

- Number of consecutive hours of sleep overnight
- Length of time to get child to sleep at bedtime (first of the night)
- Number of daytime naps
- Length of daytime naps

2. Child Sleep Health Questionnaire (CSHQ)
   The CSHQ is a parent questionnaire that has been used in many studies to examine both behavioral based and medical based sleep problems in children. The CSHQ yields a total score and eight subscale scores: 1) Bedtime Resistance, 2) Sleep Onset Delay, 3) Sleep Duration, 4) Sleep Anxiety, 5) Night Wakings, 6) Parasomnias, 7) Sleep-Disordered Breathing, and 8) Daytime Sleepiness. It has been validated in multiple groups including community children, children with diagnosed sleep disorders, children with development delay, and children with autism [17,18].

10.7 Stooling

Stooling will be measured using a newly developed stooling survey titled “Sanfilippo Stool Habit Questionnaire” which was designed based on PROMIS stooling surveys. The survey covers stool pattern (e.g. consistency, frequency, ease), stooling interference with daily activities, influence on behavior, stooling related medications, and dietary interventions.

10.8 Sanfilippo Behavior Rating Scale (SBRS)

The SBRS is a 68 item questionnaire, developed by Shapiro et al[19,24] to assess the behavioral phenotype of children with MPS III and its progression over time. There are 15 scales that rate orality, movement/activity, attention/self-control, emotional function including Anger and Fear, and Social Interaction. In addition, 12 scales are grouped into four abnormality clusters: Movement, Lack of Fear, Social/Emotional and Executive Dysfunction.

10.9 Non-communicating Children’s Pain Checklist-Revised (NCCPC-R)

The NCCPC-R is a validated scale for measuring pain in children with severe cognitive impairments[20,25]. It includes 7 scales that measure vocal, social, facial, activity, body and limbs, physiological, and eating/sleeping indicators of pain. A combined total score for pain is calculated as well.

10.10 NIH Patient Reported Outcomes Measurement Information System (PROMIS)

Per PROMIS guidance, we selected the 10 most relevant questions for parents of children with MPS III from the PROMIS Parent Proxy Fatigue item bank to make a

customized PROMIS Fatigue - Parent Proxy Custom Short Form. Customized short forms are scored using this online scoring service:
https://www.assessmentcenter.net/ac_scoringservice

10.11 Autism Parenting Stress Index (APSI)

The APSI was developed following many interviews of parents of children with autism. The Items fall into three categories: the core social disability, difficult-to-manage

behaviors, and physical issues. The APSI measures how much stress related to these three categories the parents are experiencing. The overall APSI scale score has been validated for parents of children with autism and other developmental disabilities[21].

10.12 Seizures

Seizure frequency, duration, and severity will be reported by the caregiver in real time in a study diary or by texting the research group (based on caregiver preference).

10.13 Disordered Movement

The duration, severity, and type (e.g. dystonia, chorea) of movement abnormality will be reported by the caregiver in real time for 1 week at a time in a survey. Duration will be quantified as:

- ☐ Occasional (<25% of the time)
- ☐ Intermittent (25-50% of the time)
- ☐ Frequent (50-75% of the time)
- ☐ Constant (>75% of the time)

Severity will be quantified as:

- ☐ The movement has interfered more with my child's daily activities
- ☐ The movement has interfered less with my child's daily activities
- ☐ No change in the severity of the abnormal movement

Optional: The parents can take a short, representative video of the movement disorder on the days they are recording it as above.

10.14 Individual Clinical Response (ICR)

The 5 most important clinical problems will be determined by the caregiver. Examples will be offered based on previous work[8] that include sleep disturbances, hyperactivity, frustration/impulse control/aggressive behaviors, feeding, anxiety, unhappiness, communication, social deficits, digestive issues and toileting, pain, illness/vulnerability to illness, fatigue, seizure, mobility, and gait. The caregiver will rate their top 5 outcomes on a 5-point Likert scale as follows:

0 - Not stressful

1 - Sometimes creates stress

2 - Often creates stress

3 - Very stressful on a daily basis

4 - So stressful sometimes we feel we can not cope

10.15 Gait Analysis

Gait will be filmed from 3 directions while the participant walks 30 meters and total time to complete the 30 meters will be recorded. These films will be viewed and rated by a certified pediatric physical therapist. Videos will be stored on the secure LI server indefinitely.

10.16 Vineland Adaptive Behavior Scales, Third Edition (VABS-III)

The VABS-III measures adaptive functioning in people from birth to age 90, across the domains of Communication, Daily Living Skills, and Socialization, as well as Motor Skills for the younger age ranges. Each domain is further partitioned into three more specific scales. To illustrate, the Communication domain contains Receptive, Expressive, and Written Communication. Within each scale, items describe a skill or behavior, and the respondent indicates how often the patient exhibits the skill (i.e. not whether the patient can, but whether the patient does). As the VABS-III is a norm-referenced measure based on 3,695 people from birth to 90 years, the respondent's ratings are tallied to yield scale scores, domain scores, and a total score (Adaptive Behavior Composite) that reflect the degree to which the patient is functioning within the age-typical range or has deviated from it. Age equivalent scores can be obtained for each scale; this option is particularly useful for the Motor Skills domain for which norm-based scores can be calculated only up through age 7, thus allowing for quantification of motor impairments in older patients. Test retest reliability ranges between 0.76 and 0.98 across domains[26,27]. Previous editions of the VABS have been used extensively in MPS research.

10.17 Stair Climb

A 1-flight stair climb will be filmed and evaluated for ease of climb/descent (i.e. amount of assistance, redirection, etc.) and total time required to complete stair climb and descent. Videos will be stored on the secure LI server indefinitely.

10.18 Stool Microbiome

Ongoing work in the fields of Autism and Alzheimer's Disease have investigated associations between gut dysbiosis, inflammation and neurologic/neurobehavioral changes[28,29]. We will request a stool sample at Week

-8, Day 1, Week +8, Week+16, Week +36, and Week +44 to assess for any changes in microbiota. If patients are on a probiotic supplement prior to the study, they should remain on a stable dose. Those not on probiotics will be encouraged not to begin supplementation during this trial.

10.19 Lumbar puncture and CSF collection

CSF collection will be limited to participants for whom the procedure can be conducted using only mild sedation (one agent, responds to voice; e.g. oral midazolam or lorazepam only) and without use of ultrasound or fluoroscopy.

In addition, **parents/guardians may opt out of CSF collection** even if their child meets the above criteria due to parental concerns, e.g. potential amplified negative impact of repeated sedation on the brain of their children specific to having a neurometabolic disease.

For those meeting the criteria above and whose parents/guardians consent to CSF collection, CSF will be collected at Day 1, Week 36, and Week 44 for inflammatory (IL-1β, TNF-α) and GAG (heparan sulfate non-reducing ends I0S0 and I0S6) biomarkers.

CSF will be collected using the following procedures:

1. The subject will be taken to a procedure room where equipment and medications necessary for resuscitation will be in the room. A local anesthetic (lidocaine dose as appropriate) will be administered in the skin and underlying tissue above the chosen interspace. A small spinal needle will be inserted under sterile conditions into the lumbar spinal sac. A small amount of CSF (roughly 5-8 mL) will be collected for biomarker evaluations.
2. Family members will be provided clear instructions regarding the post discharge monitoring of the subject for anticipated adverse events (AE).

10.20 Unscheduled Study Visits

Additional visits will be scheduled with local physicians as needed throughout the study period to address concerning signs or symptoms reported by subjects.

Subjects may withdraw voluntarily from the study at any time. If a subject opts to discontinue, attempts will be made to schedule and conduct an Early Termination visit for medical assessment.

If a subject withdraws after an AE, they will be followed until resolution of the issue or until their condition is stable. In the event that a subject becomes pregnant, then

discontinuation, reporting, and follow-up procedures outlined in Section 13.0 will be followed.

10.21 Safety Procedures

Contraceptive Requirements

Female subjects: Female subjects who have had menarche must agree to refrain from heterosexual intercourse or use two methods of birth control during treatment and for an additional 1 month after the last dose of anakinra. Two of the birth control methods listed below may be chosen:

- Hormonal contraception
- Male or female condoms with or without a spermicidal
- Diaphragm or cervical cap with a spermicidal
- Intrauterine device (IUD)

If a female subject is suspected of being pregnant, anakinra must be stopped immediately and may not be resumed until absence of pregnancy has been medically confirmed.

Male subjects: Male subjects are not required to use birth control during the study.

Vital signs

All subjects will be monitored after their dose on **day 1** for 6 hours and **day 2** for 4 hours with vital signs as follows:

**Screening**	After at least 5 min of rest, subject's vital signs are taken, preferably in sitting position. Vital sign measurements should be repeated and documented 3 times with at least 5 min intervals between assessments.		
	**Assessment frequency**		
**Dosing visits**	0-2 hr post-dose	2-4 hr post dose	4-6 hr post-dose
**Days 1, 2**	q 15 min (± 5 min)	q 30 min (± 5 min)	q 60 min (± 10 min)

1. Vital sign measurements are taken once per time point, preferably in a sitting position, after at least 5 minutes of rest.<br>2. Heart rate, blood pressure, and respiratory rate should be taken and recorded at each indicated time point above.<br>3. Vital signs may be monitored more frequently or for longer duration post-dose as clinically indicated.

Vitals will also be measured at Screening, Week-8, Week +8, and Week +36. They will be repeated and documented 3 times with at least 5 min intervals between assessments.

10.22 Drugs used in the research and the purpose of their use, and their regulatory approval status.

The safety and effects of anakinra treatment of patients with MPS III will be tested in this study. This medication is approved by the Food and Drug Administration (FDA) for other indications but not specifically for MPS III.

10.23 The source records that will be used to collect data about subjects

Electronic case report forms using REDCap will be used. The study site personnel will be trained on using REDCap for data entry and will enter clinical data from source documentation. Source documents will include laboratory reports, study diaries, subject records, paper surveys, etc. Any data sent electronically by email or text will be recorded on a paper source document in the subject's study chart.

10.24 What data will be collected including long-term follow-up.

No long-term follow-up data will be collected after the subject finished this 52-week study.

10.25 Subject Compensation

The study will arrange and pay directly for airfare and hotel, and provide vouchers for taxi service for travel in the Los Angeles area related to study visits, for the participant and up to two caregivers. Total travel expenses will be covered up to $2,000 per visit.

Families will also receive a gift card to compensate them for their time and the inconvenience of taking part in the study. The amount for each visit is as follows: Screening/Baseline ($320), Day 1 ($375), Week 8 ($250), and Week 36 ($250). They will receive a total of up to $1,195 if they complete the entire study. If they are unable to complete the study, the study will pay for only the visits that were completed.

10.26 *Laboratory Testing Procedures*

Specimens for analysis will be collected as described in the Schedule of Events at the time points indicated. Venipuncture will be performed by trained certified nursing assistants, nurses, nurse practitioners, or physicians and collection and handling per the manufacturer's recommendations. A central venous catheter may be utilized for all blood sampling if the patient currently has one. Samples will be collected and stored at -80°C for evaluation of other biomarkers related to inflammation, disease severity, and response to therapy.

Sample collection at weeks +2, +4, +8, +12, +16, +20, +24 (if dose increased at week +16) and +44 may be collected at LabCorp or at the participants home by phlebotomists from Phlebotek. Phlebotomists from Phlebotek will process samples and ship safety labs to Quest and the remaining samples to LI. Samples collected by Lab Corp will be process at LabCorp laboratory.

Specimen Handling and Storage

Stored Plasma, Urine and Serum Sample Directions

Plasma. Collect blood in 2 purple-top (ethylenediaminetetraacetic acid [EDTA]) tubes (4 mL each). After collection, thoroughly mix the tube by gentle inversion 8 times. Label the tube with the Subject's Study ID number. Do not write the subject's name or any other identifying information on the blood tube. Place the tube upright in a rack in the refrigerator (2 to 8°C). Centrifuge for 15 minutes at 4000 x g within 30 minutes of collection. Aliquot plasma into eight 2 mL cryovials and store samples at -80°C.

Serum. Collect blood in 2 red-top (serum separator tube [SST]) tubes (5 mL each). After collection, allow blood to clot for 30 minutes. Label the tube with the Subject's Study ID number. Do not write the subject's name or any other identifying information on the blood tube. Place the tube upright in a rack in the refrigerator (2 to 8°C). Centrifuge for 15 minutes at 4000 x g within 30 minutes of collection. Aliquot serum into five 2 ml cryovials and store samples at -80°C.

Urine. Aliquot 2 mL into each of 8 different cryovials labeled "Urine".

Whole Blood. Collect 1 Cell Preparation Tube (CPT) with Sodium Heparin tube (8 mL). Label the tube with the Subject's Study ID number, study visit, date of visit, and "whole blood". Do not write the subject's name or any other identifying information on the blood tube. Keep at room temperature until pick up by Dr. Anna Luzzi or Dr. Michelina Iacovino.

RNA. Prior to specimen collection: Confirm that informed consent for genetic testing has been obtained prior to the collection of any specimens.

Specimen collection: One 2.5 ml PAXgene tube (RNA extraction)

Invert 8-10 times immediately after drawing

Maintain tubes in upright position at room temperature for a minimum of 2 hours

After 2 hours at room temp, place the PAXgene tube upright in a wire rack to allow for adequate air circulation during freezing in the -20ºC freezer for 24 hr. (NOTE: This must be manual defrost model – not a frost-free model.) Do not use Styrofoam as the tubes tend to stick to the foam and may crack.

Store in -80□C LP#1 freezer for long-term storage.

All specimens must be labeled with a code. Unlabeled specimens will be destroyed.

All specimens must be accompanied by a completed lab sheet.

11.0 Data and Specimen Banking

Stored samples will be identified by an indirect identifier (study ID) indefinitely. The study investigators and study coordinators will have access to the link between subject information and study ID. The link between subject information and their study ID will be destroyed 15 years after the study closes.

If stored samples are used in the future to look at new markers the subject and guardian will not be notified nor will they receive the results from the analysis. If the samples are used by other research groups for analysis, the subject and guardian will not be notified. However, any samples that are sent to another researcher will be identified only with a number and will not be able to be traced back to the subject. The subject will not be identified in any publication or report from this study. The subject or guardian can request that blood, urine, and DNA samples be destroyed at any time, prior to 15 years after the study closes, by contacting the study PI.

12.0 Data Management and Confidentiality

12.1 Subject Population(s) for Analysis

- Protocol-compliant population (PP): Any subject who received at least 85% of planned doses and attended all scheduled study visits.
- Safety population (SP): All subjects who receive any amount of treatment.

Safety population (SP) will be used for the primary analyses and the secondary analysis would be based on protocol-compliant population (PP) as part of sensitivity analyses.

12.2 Analysis

Primary objective

Outcome variables include AEs/tolerability, 2 most bothersome symptoms, and multi-domain responders index (MDRI) as described in section 9.1. Binary AEs such as hospitalization (≥1) will be summarized using frequencies/rates by the two period(pre-observation, treatment). The multiple event such as the total number of seizures will be summarized using median with the inter-quartile range (IRQ). For testing changes between the two periods, the McNemar's test (binary event) or Wilcoxon Singed Rank test (multiple events and MDRI) will be used. For continuous outcomes such as laboratory results, a paired t-test and a repeated measures ANOVA will be used. Residual analyses will be conducted to check the model assumption. If it violates assumption, Friedman test (nonparametric) will be used instead.

Secondary objective

Outcome variables include Individual Clinical Response (ICR, 0~25: sum of the top 5 domains on a 5-point Likert scale), Gait analysis results (improved, no change, worsened), Stair climb/descent (time and quality of improvement), Sleep diary (sum of the number of overnight awakenings, average duration of overnight awakenings, and duration of time for initial sleep onset at bedtime), and Stooling survey results. For univariate analyses, similar statistical tests will be used as ones used for the primary objective, depending on the data type of the outcome for comparing two study periods. For multivariate analyses, mixed effect models (linear for continuous outcomes and non-linear such as multinomial logistic regression for categorical outcomes and Poisson (or Negative Binomial regression model , if over-dispersion is apparent) for count outcomes) will be used using 2-4 different time points (pre-observation, 8, 36 week treatment, post-observation periods) as within subject factor. Covariates such as sex and age will be considered in multivariate models if they are found to be statistically significant in changes of outcomes. Sub-scale analyses will be conducted if changes in the composite score are found to be statistically significant or are reached to a clinically meaningful boundary. We expect to have full data without any missing for all outcomes. However, in case of missing data, two imputation methods, i) last observation forward, ii) multiple imputation assuming missing at random, will be considered.

Exploratory objective

Explanatory outcomes include stool microbiome and Vineland 3. The similar analyses as ones for primary and secondary objectives will be carried out based on data types.

Analyses will be conducted using the programs of SAS v9.4 (SAS Institute, Cary, NC). All tests will be two-tailed and a P value less than 0.05 will be considered statistically significant. The primary outcome will be tested at the 5% significance level. Secondary, exploratory and sensitivity analyses will not be adjusted for multiplicity. Therefore, the results will be interpreted with caution and discussed with recognition of inflated family-wise type I error rate.

Descriptive statistics will be provided on all study data collected from the total cohort (N=20) from the entry to the trial to the completion of 44 weeks follow-up continuous variables will be summarized as numbers of observed and missing values, mean, standard deviation, median, minimum and maximum. Categorical variables will be described as frequencies and percentages.

Any post-hoc, exploratory analyses or deviations which were not identified in this data analysis plan but are completed to support the planned analyses will be clearly identified and the reasons documented.

12.3 Power

The sample size of 20 for the study was determined primarily by feasibility rather than statistical power due to the extremely rare nature of the disease. However, the level of precision in estimation of AEs during the treatment period was computed with a given samples size of 20 using 95% confidence interval. For estimation of the binary event, the margin of the error would be 13% to 18% when the sample proportion are assumed to be 5% to 15%. For continuous outcomes, the margin of the error would be a half fold of the standard deviation in estimation of paired difference between the treatment and first observation period.

12.4 Database

All study visit data will be stored using REDCap (https://www.project-redcap.org). Ranges and logic reviews of the data will be performed. Access to the REDCap database will be granted over a secure web connection with authentication and data logging. Data from REDCap will be extracted, cleaned and merged into a single study dataset (stored in SAS v 9.4 for 64-bit) and saved under the secured institutional network drive and used by the study statistician Dr. Pak for all data analyses.

12.5 Data Quality Control

Upper and lower limits will be included in the REDCap database to flag any entries that are not within the expected range.

13.0 Provisions to Monitor the Data to Ensure the Safety of Subjects

The plan for ensuring subject safety will include the following elements:

1. Clinical procedures will only be performed by properly trained personnel who are qualified by training and licensure to perform the procedures.

2. Investigator and all study personnel have completed required training regarding Human Subject Protections. All personnel will comply with all related regulations and laws.

3. Patients will be rigorously screened against inclusion/exclusion criteria to ensure that their participation is safe.

4. Adverse events and SAEs will be assessed and followed throughout study. Subjects will have contact information to enable them to contact study personnel easily and quickly.

5. Study data and information will be kept confidential and managed in accordance with requirements of Health Insurance Portability and Accountability Act of 1996 (HIPAA). All data will be stored in locked offices and not released without subject permission.

6. Subject may discontinue participation at any time, for any reason. Any subject who fails to complete the research procedures within acceptable parameters, or is unable to safely tolerate participation in the study will be withdrawn.

7. Institutional Review Board approval and IND approval will be obtained.

8. Monitoring will be conducted by the research team, institutional review board (IRB) and independently (i.e. at a minimum of annually) by qualified staff of Harbor-UCLA Clinical and Translational Science Institute (CTSI) in accordance with the established Monitoring Plan.

9. The PI will allocate adequate time for such monitoring activities and will ensure that the monitor or other compliance or quality assurance reviewer is given access to all study-related documents and study related facilities (e.g. pharmacy, diagnostic laboratory, etc.) and has adequate space to conduct the monitoring visit.

13.1 Additional Provisions to Monitor Genetic Data

Genetic studies have raised concern as to whether the studies would place research subjects at risk for discrimination based on genetics. The federal Genetic Information Nondiscrimination Act (GINA) was passed to address this concern. GINA makes it illegal for medical insurance companies and most employers to discriminate based on genetic information. The protections of GINA do not apply to life, disability, or long-term-care insurance.

To make the best possible use of GWAS and data from large-scale genomics technologies such as sequencing, methylation and expression, the National Institutes of Health (NIH) policy requires researchers to share the information from GWAS studies with other researchers. When we perform a GWAS or other high throughput genotyping and sequencing, the data will be shared for research purposes through the NIH Genome-Wide Association Study database at the National Center for Biotechnology Information (NCBI), part of the National Library of Medicine. This is a national health research database that allows broad sharing of data to qualified investigators. All qualified investigators are required to obtain authorization from NIH to gain access to this database. All information that is shared, will be identified by a code number, and all other identification will be removed.

Once submitted, the NIH will control these data. The NIH is committed to protecting the confidentiality of all the information it receives, but will also comply with relevant laws that might include Freedom of Information Act (FOIA) requests for non- identifiable information.

However, there has been some concern that there may still be ways that research subjects' privacy might be at risk. As a result, the NIH has made access to the shared information more restricted to assure that privacy is protected. Researchers who wish to use information from this database must request access and explain how and for what purpose they will use the information. Researchers and the administration at their institution must promise to protect confidentiality and only use the information for the purposes included in the request form. They must also provide assurances that data security measures are in place to prevent misuse of the information. The NIH has security measures in place to secure all data submitted to the repository, however security breaks are possible since data is stored in an electronic format. The NIH may make more changes in the future with their data access policy.

13.2 Definitions for recording/reporting adverse events

Adverse Event

An AE is any symptom, sign, illness or experience that develops or worsens in severity during the course of the study. Intercurrent illnesses or injuries will be regarded as AEs. Abnormal results of laboratory or diagnostic procedures are considered to be AEs if the abnormality:

- Results in study withdrawal
- Is associated with a serious adverse event (SAE)
- Is associated with clinical signs or symptoms
- Leads to additional treatment or to further diagnostic tests
- Is considered by the Investigator to be of clinical significance

Adverse Reaction

An adverse reaction is any AE caused by a drug. Adverse reactions are a subset of suspected adverse reactions.

Suspected Adverse Reaction

A suspected adverse reaction is an AE for which there is a reasonable possibility that the drug caused the AE.

Serious Adverse Event (SAE)

An SAE is any AE that is:

- Fatal
- Life-threatening
- Requires or prolongs a hospital stay
- Results in persistent or significant disability or incapacity
- A congenital anomaly or birth defect

Important medical events are events that may not be immediately life-threatening, but are clearly of major clinical significance and may be SAEs. They may jeopardize the subject, and may require intervention to prevent one or the other serious outcomes noted above.

Hospitalization

Hospitalization shall include any initial admission (even if less than 24 hours) to a healthcare facility as a result of a precipitating clinical adverse effect; to include transfer within the hospital to an intensive care unit. Hospitalization or prolongation of hospitalization in the absence of a precipitating, clinical adverse effect (e.g. for a preexisting condition not associated with a new adverse effect or with a worsening of the preexisting condition; admission for a protocol-specified procedure) is not, in itself, a serious adverse effect.

Expected Adverse Event

Expected AEs are those that are known to be associated with or have the potential to arise as a consequence of participation in the study. This will include any AE or SAE that are expected consequences of having MPS . These expected events include but are not limited to, injection site reactions, injection site pain, headache, seizures, symptoms of movement disorder, sinusitis/ear infections, upper respiratory infection, fevers, nausea, vomiting, diarrhea, skin rash, pain, cardiac valve insufficiency, obstructive sleep apnea, kyphoscoliosis, genu valgum, cervical cord compression, hearing loss, in-toeing, osteonecrosis of the femoral head, retinitis pigmentosa with vision impairment, carpal tunnel syndrome, and trigger fingers.

Unexpected Adverse Event

An AE or suspected adverse reaction is considered unexpected if it is not listed in the drug package insert or Protocol at the specificity or severity that has been observed.

Unanticipated Problems Involving Risk To Subjects or Others (UPIRTSO)

An AE that in the opinion of the PI is unexpected, related to the drug, and serious.

13.3 Recording of Adverse Events

At each contact with the subject, the Investigator must seek information on AEs by specific questioning and, as appropriate, by examination. Information on all AEs should be recorded immediately in the source document, and also in the appropriate AE module of the case report form (CRF). All clearly related signs, symptoms, and abnormal diagnostic procedures results should be recorded in the source document, though should be grouped under 1 diagnosis.

All AEs occurring during the study period must be recorded. The clinical course of each event should be followed until resolution, stabilization, or until it has been determined that the study treatment or participation is not the cause. Serious adverse events that are still

ongoing at the end of the study period must be followed up to determine the final outcome. Any SAE that occurs after the study period and is considered to be possibly related to the study treatment or study participation should be recorded and reported immediately.

Toxicity and AEs will be classified according to the National Cancer Institute (NCI) Common Toxicity Criteria (CTC) V 4.1 and causality will be classified as:

Unrelated - The AE is likely NOT related to study treatment or procedures

Related - The AE is likely related to study treatment or procedures

13.4 Study Stopping Rules

This study may be terminated by the PI, IRB, Safety Monitor or FDA at any time. Reasons for terminating the study may include the following:

- The incidence or severity of AEs in this or other studies indicates a potential health hazard to subjects.
- Subject enrollment is unsatisfactory.
- Data recording is inaccurate or incomplete.
- Adverse events which may result in study termination include, but are not limited to:
  - Death
  - Cancer
  - Serious infection
  - Anaphylaxis
  - Acute hepatic failure
- ≥ 2 patients develop the same CTCAE Grade 3.
- 1 patient develops a CTCAE Grade 4 or higher.

13.5 Reporting of Serious Adverse Events

Study Sponsor Notification by Investigators

An SAE must be reported to the study Sponsor by telephone or fax (preferred) within 24 hours of the event. An SAE Form must be completed by the Investigator and faxed to the study Sponsor within 24 hours. The Investigator will keep a copy of this SAE form on file at the study site. Report SAEs by phone and fax to:

Lynda Polgreen, MD, MS 310-222-1961 (telephone)
310-972-2962 (fax)

At the time of the initial report the following information should be provided:

- Study Identifier
- Study Center
- Subject Number
- A description of the event
- Date of onset
- Current Status
- Whether study treatment was discontinued
- The reason why the event is classified as serious
- Investigator assessment of the association between the event and study treatment

Within the following 48 hours, the Investigator must provide further information on the SAE in the form of a written narrative. This should include a copy of the completed SAE Form, and any other diagnostic information that will assist the understanding of the event. Significant new information on ongoing SAEs should be provided promptly to the study Sponsor.

IRB Notification by Investigators

Reports of all SAEs (including follow-up information) must be submitted to the IRB within 10 working days if it falls under the UPIRTSO guidelines (unexpected and related to study drug). All deaths, regardless of expectedness or causality, must be reported to the IRB within 10 working days. Copies of each report and documentation of IRB notification and receipt will be kept in the Clinical Investigator's binder.

FDA Notification by Sponsor

The study Sponsor shall notify the FDA by telephone or by fax (preferred) transmission of any unexpected SAE associated with the use of the study drug for which the Investigator holds an active IND as soon as possible but no later than 7 calendar days from the Sponsor's original receipt of the information.

If a previous AE that was not initially deemed reportable is later found to fit the criteria for reporting, the study Sponsor will submit the AE in a written report to the FDA (as applicable) as soon as possible, but no later than 15 calendar days from the time the determination is made.

UPIRTSO Events

Investigators are required to submit a report of UPIRTSO events to the IRB within 10 working days of first learning of the event.

Pregnancy

If a female subject becomes pregnant during the study, she will be withdrawn from the study immediately. Though pregnancy is not by definition an SAE, it will be subject to SAE reporting requirements described in Section 13.5.

Though the subject will be withdrawn from the study, permission to record survival and adverse event (malignancy, hospitalization for infection) data for the mother to the end of the pregnancy and of survival data for the baby at birth will be requested.

14.0 **Withdrawal of Subjects**

Subjects will be encouraged to complete the study; however, they may voluntarily withdraw at any time. If a subject is withdrawn prior to study completion, attempts will be made to schedule and conduct an Early Termination visit for medical assessment. If a subject cannot be contacted after 5 telephone calls and 3 letters/emails, they will be considered lost to follow-up.

Subjects who discontinue study medication (e.g. due to non-compliance, AEs, etc.) will be asked to remain in the study for follow-up per the study schedule to preserve the ability to perform an intent to-treat (ITT) analysis. If a subject or their legal guardian (as applicable) withdraws consent to participate in the study, attempts will be made to obtain permission to record survival and adverse event (malignancy, hospitalization for infection) data at the Week +44 study point.

Study drug will be discontinued in any subject meeting the following criteria for drug-induced liver injury:

- ALT or AST > 8x upper limit of normal (ULN)
- ALT or AST > 5x ULN for more than 2 weeks
- ALT or AST >3x ULN and (total bilirubin >2x ULN or INR > 1.5)
- ALT or AST >3x ULN with the appearance of fatigue, nausea, vomiting, right upper quadrant pain or tenderness, fever, rash, and/or eosinophilia (>5%)

Management of suspected drug-induced liver injury will be consistent with the FDA Guidance for Industry Drug-Induced Liver Injury: Premarketing Clinical Evaluation. Specifically, an increase of ALT or AST of>3x ULN will be followed by repeat testing within 48-72 hours of ALT, AST, alkaline phosphatase, and total bilirubin level. There will be inquiry made about symptoms. If symptoms persist or repeat testing shows ALT or AST >3x ULN close observation to determine whether the abnormalities are improving or worsening will be initiated as follows:

- Repeating liver enzyme and serum bilirubin tests two or three times weekly. Frequency of retesting can decrease to once a week or less if abnormalities stabilize or the trial drug has been discontinued and the subject is asymptomatic.
- Obtaining a more detailed history of symptoms and prior or concurrent diseases.
- Obtaining a history of concomitant drug use (including nonprescription medications and herbal and dietary supplement preparations), alcohol use, recreational drug use, and special diets.
- Ruling out acute viral hepatitis types A, B, C, D, and E; autoimmune or alcoholic hepatitis; NASH; hypoxic/ischemic hepatopathy; and biliary tract disease.
- Obtaining a history of exposure to environmental chemical agents.
- Obtaining additional tests to evaluate liver function, as appropriate (e.g. INR, direct bilirubin).
- Considering gastroenterology or hepatology consultations.

Study drug will be discontinued in any subject developing one of the following:

- Serious infection (e.g. cellulitis, pneumonia requiring hospitalization, or bone and joint infections, from fungal, mycobacterial and bacterial pathogens; fungal sinusitis)
- Malignancies (e.g. Lymphomas, breast, respiratory, and digestive system cancers)
- Severe hypersensitivity reactions including anaphylactic reactions and angioedema

15.0 Risks to Subjects

15.1 Venipuncture

The risks of venipuncture are transient pain, bleeding, lightheadedness, bruising, possible vasovagal reaction, and infection. Only trained certified nursing assistants, nurses, nurse practitioners, or physicians will be allowed to perform venipuncture to minimize the risk of complication.

15.2 Anakinra

The following have been reported as adverse reactions related to anakinra in other studies:

- Serious infections (e.g. Cellulitis, pneumonia, sinusitis, upper respiratory tract infections, ear infections, nasopharyngitis, gastritis, bone and joint infections, from fungal, mycobacterial and bacterial pathogens)
- Injection-site reactions (e.g. Erythema, ecchymosis, inflammation, and pain)
- Malignancies (e.g. Lymphomas, breast, respiratory, and digestive system cancers)
- Hematologic events (e.g. Decreased total white blood cell counts, neutropenia, eosinophilia, thrombocytopenia, epistaxis)
- Hypersensitivity reactions including anaphylactic reactions, angioedema, urticaria, rash, and pruritis
- Immunogenicity (i.e. Anti-anakinra binding antibodies)
- Headache, dizziness
- Nausea, diarrhea, abdominal pain
- Arthralgia
- Flu-like symptoms; pyrexia, malaise

16.0 Potential Benefits to Subjects

Subjects will be treated with anakinra. These treatments have potential direct benefits to the subjects. These potential direct benefits include, but are not limited to, improved behavior, sleep, stooling, communication, mood, and gait; as well as decreased seizure frequency, disordered movement and fatigue.

17.0 Sharing of Results with Subjects

17.1 Individual subject safety laboratory results that are clinically significant, as determined by the study PI, will be shared with subject's parent/guardian and the subject's primary care physician by email or phone/fax if email is not available. Confirmation of receipt of these results will be required either by email or phone call.

17.2 At the end of the study (Week +44), all results will be given to subjects/parents/guardians upon request.

18.0 Setting

Study visits will take place at the LI Clinical and Translational Research Center (CTRC).

19.0 Resources Available

This study will be conducted by the Rare Disease Clinical Research Group (RDCRG) at LI. This group includes four physicians, two study coordinators, one regulatory specialist, and one program coordinator, who all have previous experience conducting clinical research in MPS and other rare disease, pediatric and adult, populations. They are thus knowledgeable of the local study site, culture, and society. All are current with their education/training requirements for conducting research in human volunteers.

The CTRC provides support and resources for studies on human research participants. Research nursing within the CTRC integrates high quality patient care, patient-oriented research, and education. CTRC nurses support protocol implementation and the research subject's participation in clinical trials. They provide skillful nursing care, expertise in protocol implementation and data collection. The nurses have diverse backgrounds, experiences, and they hold certifications in a variety of specialized areas such as bone densitometry, cardiac monitoring, and advanced cardiac life support.

All study personnel interacting with subjects are certified in the conduct of clinical trials, good clinical practice, the protection of human subjects, and HIPAA regulations to assure compliance with regulatory requirements. They are also trained to ensure that research participants are fully informed about the details of their research protocol visits, protection of private medical health information, and the Research Subject's Bill of Rights.

To assist the investigator and the research subject during the consenting process and on follow-up visits, several of our staff members can provide translation in Spanish, Tagalog, and other languages. The outpatient unit contains nine exam rooms (3 with hospital beds), 2 consultation rooms, and 1 multipurpose room.

Medical or psychological resources are available through Harbor-UCLA Medical Center.

The PI meets on a weekly basis with the RDCRG and will conduct an in-service training session with CTRC staff to ensure that all persons assisting with the trial are adequately informed about the protocol, the investigational product, and their trial-related duties and functions.

Funding for this study will be provided by the Cure Sanfilippo Foundation. Study drug will be provided by Sobi. The UCLA CTSI Grant UL1TR001881-01 will provide partial funding for costs related to utilization of CTSI facilities and resources.

20.0 Prior Approvals

Prior approval for this study will be obtained from the LI Human Subject Committee and the LI CTSI/CTRC.

21.0 Recruitment Methods

21.1 Potential subjects will be recruited through the study physicians' and other MPS physicians' clinical practices, as well as advertisements on MPS foundation websites including that of the Cure Sanfilippo Foundation and the ConnectMPS patient registry. The study will be registered on clinicaltrials.gov. Information on the study will be shared through social media as well. For out-of-state participants, the study will be initially discussed by phone and, if they are interested in participation, we will combine the Screening and Week -8 visits, getting written consent/assent at the beginning of the visit. Patients and/or their parents or legal guardians will be given the option of receiving copies of the consent/assent by email, mail, or fax for further review prior to their Screening/Week -8 visit. The study coordinator will then contact the patient or parents/legal guardians within a few days to schedule a Screening/Week -8 visit if they are interested in participating.

22.0 Local Number of Subjects

About 20 subjects will take part in this study.

23.0 Provisions to Protect the Privacy Interests of Subjects

Study data and information will be kept confidential and managed in accordance with requirements of Health Insurance Portability and Accountability Act of 1996 (HIPAA). All data will be stored in locked offices and not released without subject permission.

Subjects will interact with and provide personal information to only individuals who are on the study site delegation log or employees of the CTRC. All individuals interacting

with study subjects and their caregivers will have successfully completed HIPAA training.

The research team will obtain Authorization for Release of Protected Health Information (PHI) from the subject or subject's parent or guardian to permit them to access medical information about the subjects.

24.0 Compensation for Research-Related Injury

Subjects will get medical treatment if they are injured as a result of taking part in this study. The cost of this medical treatment will be the responsibility of the subject/subject's parent/guardian. The cost will not be covered by the study.

25.0 Economic Burden to Subjects

Subjects will not be responsible for any costs directly related to their participation in this study. Indirect costs as a result of participation in this study, such as loss of work days, will be the responsibility of the subject or the subject's parent or guardian.

26.0 Consent Process

Each subject, subject's parent, or subject's legal guardian will be provided a consent form describing this study and providing sufficient information to make an informed decision about their participation in this study. The consent form will be submitted with the protocol for review and approval by the IRB for the study. The formal consent of an adult subject or minor subject's parent/legal guardian, using the IRB-approved consent form, must be obtained before that subject is submitted to any study procedure. This consent form must be signed by the subject or parent/legal guardian, and the Investigator-designated research professional obtaining the consent. A blank copy of the IRB-approved form must be kept on-site by the Investigator.

Assent must be obtained from all minor subjects aged 7 to 17 years who are cognitively able to give assent, in addition to the informed consent of the parent/legal guardian in accordance with Federal Regulations and the IRB.

26.1 Subjects who are not yet adults

Subjects in the United States are considered minors if they are 17 years or younger, with the exception of Alabama (19 years), Nebraska (19 years or upon marriage), and Mississippi (21 years).

Parental permission will be obtained for minors from one parent even if the other parent is alive, known, competent, reasonably available, and shares legal responsibility for the care and custody of the child.

Parental or guardian (as defined by Department of Health and Human Service [DHHS] and FDA regulations) consent will be obtained. A guardian must provide written documentation of the legal ability to consent to for the child's participation in research. A copy of this documentation will be kept with the consent document.

26.2 Cognitively Impaired Adults /Adults Unable to Consent

The individuals from whom permission will be obtained are the following in descending order of priority:

- The person's agent pursuant to an advance health care directive
- The conservator or guardian of the person having the authority to make health care decisions for the person
- The spouse of the person
- An individual as defined in Section 297 of the Family Code
- An adult son or daughter of the person
- A custodial parent of the person
- Any adult brother or sister of the person
- Any adult grandchild of the person
- An available adult relative with the closest degree of kinship to the person

26.3 The process for assent of the subjects

Assent will be attempted for all subjects age 7-17 years and for cognitively impaired adults. The process for assent will include reviewing the assent document with the subject and their parent/guardian in a verbal face-to-face discussion, asking them for questions and discussing these, then asking the subject to sign and date the assent document if they would like to participate in the study.

Assent will be documented in a progress note.

Should a child enroll at the age of 5 years, assent will be obtained once the child is 7 years old and capable of providing it. Should a child enrolled in the study reach 18 years during the study, consent will be obtained from them at that time.

26.4 Process to Document Consent in Writing

We will be following "SOP: Written Documentation of Consent (HRP-091)"

26.5 Vulnerable Populations

We will include the following special populations:

- Adults unable to consent – yes, a separate consent has been prepared for the guardian of these adult subjects.
- Individuals who are not yet adults (infants, children, teenagers) – yes, a separate consent has been prepared for the parent/guardian of these pediatric subjects as well as an assent form that will be completed with all pediatric subjects age 7 and older.
- Pregnant women - no
- Prisoners – no

27.0 **Specific Drug Supply Requirements**

27.1 Drug Receipt

Kineret™ is supplied in single-use preservative free, prefilled glass syringes with 27-gauge needles. Each prefilled glass syringe contains 100 mg of anakinra per 0.67 mL. The full syringe contains 100 mg anakinra. Anakinra will be ordered by the Investigational Drug Service (IDS) at LI. Study drug inventory will be documented.

27.2 Packaging

The immediate package of an investigational drug intended for human use shall bear a label with the statement:

Caution: New Drug--Limited by Federal law to investigational use.

27.3 Storage

Vials of anakinra will be stored at 2-8°C, protected from light.

27.4 Dispensing of Study Drug

Drug will be provided by Sobi and dispensed by the LI IDS, who are experienced in the safe and proper handling of study drug. Medication will be dispensed in accordance with the protocol. The Investigator and IDS will keep

accurate records of the medications, the amount dispensed to and returned by the patients, and the disposition at the end of the study.

27.5 Parent Training

Parents will be trained on administration of the study drug during their Day 1 study visit and required to independently administer the injection on Day 2 before being discharged home. Parents will then administer the drug daily at home.

Parents will be educated on signs and symptoms of anaphylaxis and instructed on procedures for obtaining local medical care in the event of anaphylaxis (e.g. oral diphenhydramine, IM epinephrine, and call 911) or other study drug related concerns (contact study MD on call, local MD, or go to local ED or Urgent Care Clinic). Diphenhydramine and epinephrine will be dispensed to the families to take home with them, with instructions on the use of these for treatment of allergic reactions including anaphylaxis.

27.6 Return or Destruction of Study Drug

At the completion of the study, there will be a final reconciliation of study agents that were shipped, consumed, and remaining. This reconciliation will be logged on the drug reconciliation form, signed and dated. Any discrepancies noted will be investigated, resolved, and documented prior to return or destruction of unused study drug. Drug destroyed on site will be documented in the study files. The Investigational Pharmacy will be responsible for the destruction of medication at conclusion of the study or upon drug expiration.

27.7 Dosing

In this study anakinra subcutaneous (SC) will be administered at a dose level of 100 mg SC once daily. Anakinra will be administered every other day in patients who have severe renal insufficiency or end stage renal disease (defined as creatinine clearance < 30 mL/min as estimated from serum creatinine levels).

Dose will be increased to 200 mg SC once daily, with a maximum dose limit of 8 mg/kg/day, at **Week +8** if the change in at least one of the two most bothersome outcomes selected by the parents/guardians at the Day 1 visit has not improved by ≥ the MCID as defined in Section 9.1. If the MCID is not achieved after 8 weeks on maximum trial dose of 200mg (max dose 8 mg/kg/day), **treatment with anakinra may be discontinued after a review and discussion between the study PI and subject's parent(s) of their child's individual results from all study outcome measures**.

Dose will be increased to 200 mg SC once daily, with a maximum dose limit of 8 mg/kg/day, at **Week +16** if the change in at least one of the two most bothersome outcomes selected by the parents/guardians at the Day +1 visit have worsened by ≥ the MCID as defined in Section 9.1 between week +8 and week +16, after an improvement from Day +1 to week +8. If the MCID is not achieved after 8 weeks on maximum trial dose of 200mg (max dose 8 mg/kg/day), **treatment with anakinra may be discontinued after a review and discussion between the study PI and subject's parent(s) of their child's individual results from all study outcome measures**.

For example:
- At week +8, if a subject has not improved in the chosen items then the dose will be increased to 200 mg SC daily.
- At week +8, if a subject has improved, then the dose stays at 100 mg SC daily. However, if this individual then has worsening of the two most bothersome outcomes from week +8 to week +16, after improving from day 1 to week +8, the dose will be increased to 200 mg SC daily at that point (i.e. increase at week +16 after not increasing at week +8).

Dose will be decreased by 50 mg SC once daily at any time throughout the study if a participant develops any of the following:

- Neutropenia <1200 cells/microliter persistent for ≥2 weeks
- Thrombocytopenia <50 x 10^9 platelets/liter persistent for ≥2 weeks
- Mild/moderate hypersensitivity reactions including urticaria, rash, and pruritis
- Participants develops a CTCAE grade 3 AE

Anakinra will be stopped if the AE above does not resolve within 2 weeks of decreasing the dose by 50 mg SC once daily. Anakinra will be restarted at 0.5 times the last dose administered once the AE is resolved. If the AE recurs with restarting anakinra, it will be discontinued and the subject monitored per protocol.

The starting dose is the same dose level as recommended for adult rheumatoid arthritis patients. The dose escalation is within the range of the recommended dose in Neonatal onset multisystem inflammatory disease (NOMID), including also pediatric patients, is related to body-weight, with a recommended starting dose of 1-2 mg/kg up to a maximum dose of 8 mg/kg.

Smaller children with lower body weight need higher dose levels/kg body-weight in order to achieve similar anakinra serum exposure as in older, heavier children, due to a higher capacity to eliminate anakinra/kg body-weight, based on pharmacokinetic data in 22

systemic-onset juvenile idiopathic arthritis (SJIA) pediatric patients in the age range 2-17 years.[30]

As an example, based on this study, a patient with a body-weight of 20 kg is predicted to require a dose of 4 mg/kg/day to achieve similar anakinra exposure (area under the curve $[AUC]_{0\text{-}24h,\ ss}$ and $C_{min,\ ss}$) as a dose of 100 mg in a 50 kg patient. Additional examples are given below:

Predicted exposure of anakinra in three hypothetical patients

	Anakinra 2 mg/kg/day (max 100 mg)		Anakinra 4 mg/kg/day (max 200 mg)	
**BW**	**AUC0-24h,ss* (ng·h/mL)**	**Cmin,ss* (ng/mL)**	**AUC0-24h,ss* (ng·h/mL)**	**Cmin,ss* (ng/mL)**
**20 kg**	11561	95	23121	191
**50 kg**	18776	236	37552	472
**100 kg**	13556	212	27111	424

* Predicted steady-state exposure based on the following published population PK parameters for anakinra (Urien et al 2013): $k_a = 0.38\ (h^{-1})$; $CL/F = 0.847 \times (BW/70)^{0.47}\ (L/h)$; $V/F = 2.581 \times (BW/70)^{0.76}\ (L)$

The patients to be included in the current study will be age 4 years and older. Given that the pharmacokinetics of anakinra in children support a higher dose level/kg body-weight the same daily dose 100 mg anakinra is planned for this study. The planned daily anakinra dose 100 mg will be within the recommended dose range for anakinra in pediatric patients.

27.8 Investigational New Drug Application

This study will be conducted under an IND per FDA Regulations Title 21 CFR 312. Electronic signatures or records will not be used (Title 21 CFR 11). All investigators and staff who are listed on the study delegation log will report their financial disclosures per LI SOP and these disclosures are reviewed by the LI Conflicts of Interest Committee to assist with management of any significant financial conflicts of interest (Title 21 CFR 54). FDA approved study drug will be supplied by Sobi using Good Manufacturing Practice (Title 21 CFR 210-211).

27.9 Publication Plan

Neither the complete nor any part of the results of the study carried out under this protocol, nor any of the information provided by the Sponsor for the purposes of performing the study, will be published or passed on to any third party without the consent

of the study Sponsor. Any Investigator involved with this study is obligated to provide the Sponsor with complete test results and all data derived from the study.

28.0 References

1. Cleary, M. A. & Wraith, J. E. Management of mucopolysaccharidosis type III. *Arch. Dis. Child.* **69**, 403–406 (1993).
2. Shapiro, E. G. *et al.* A Prospective Natural History Study of Mucopolysaccharidosis Type IIIA. *J. Pediatr.* **170**, 278-287.e1–4 (2016).
3. Rumsey, R. K. *et al.* Acquired autistic behaviors in children with mucopolysaccharidosis type IIIA. *J. Pediatr.* **164**, 1147-1151.e1 (2014).
4. Potegal, M. *et al.* Mucopolysaccharidosis Type IIIA presents as a variant of Klüver-Bucy syndrome. *J. Clin. Exp. Neuropsychol.* **35**, 608–616 (2013).
5. Héron, B. *et al.* Incidence and natural history of mucopolysaccharidosis type III in France and comparison with United Kingdom and Greece. *Am. J. Med. Genet. A.* **155A**, 58–68 (2011).
6. Grant, S. *et al.* Parental social support, coping strategies, resilience factors, stress, anxiety and depression levels in parents of children with MPS III (Sanfilippo syndrome) or children with intellectual disabilities (ID). *J. Inherit. Metab. Dis.* **36**, 281–291 (2013).
7. Shapiro, E. G., Lockman, L. A., Balthazor, M. & Krivit, W. Neuropsychological outcomes of several storage diseases with and without bone marrow transplantation. *J. Inherit. Metab. Dis.* **18**, 413–429 (1995).
8. O'Neill, C., Porter, K., Drake, E. & Peay, H. L. Meaningful treatment outcomes for Sanfilippo syndrome: A study of caregiver preferences and prioritization. *Mol. Genet. Metab.* **126**, S112–S113 (2019).
9. Polgreen, L. E. *et al.* Elevated TNF-α is associated with pain and physical disability in mucopolysaccharidosis types I, II, and VI. *Mol. Genet. Metab.* (2016). doi:10.1016/j.ymgme.2016.01.012

10. Archer, L. D., Langford-Smith, K. J., Bigger, B. W. & Fildes, J. E. Mucopolysaccharide diseases: a complex interplay between neuroinflammation, microglial activation and adaptive immunity. *J. Inherit. Metab. Dis.* **37**, 1–12 (2014).

11. Simonaro, C. M. Cartilage and chondrocyte pathology in the mucopolysaccharidoses: The role of glycosaminoglycan-mediated inflammation. *J. Pediatr. Rehabil. Med.* **3**, 85–88 (2010).

12. Bigger, B. W. *et al.* Interleukin-1 plays a central role in behaviour abnormalities in mucopolysaccharidosis type III (MPS III). *Mol. Genet. Metab.* **123**, S24–S25 (2018).

13. Parker, H. & Bigger, B. W. The role of innate immunity in mucopolysaccharide diseases. *J. Neurochem.* **148**, 639–651 (2019).

14. Holley, R. J. *et al.* Macrophage enzyme and reduced inflammation drive brain correction of mucopolysaccharidosis IIIB by stem cell gene therapy. *Brain J. Neurol.* **141**, 99–116 (2018).

15. Brands, M. M. G. *et al.* Pain: a prevalent feature in patients with mucopolysaccharidosis. Results of a cross-sectional national survey. *J. Inherit. Metab. Dis.* **38**, 323–331 (2015).

16. Polgreen, L. E. *et al.* Pilot study of the safety and effect of adalimumab on pain, physical function, and musculoskeletal disease in mucopolysaccharidosis types I and II. *Mol. Genet. Metab. Rep.* **10**, 75–80 (2017).

17. Owens, J. A., Spirito, A. & McGuinn, M. The Children's Sleep Habits Questionnaire (CSHQ): psychometric properties of a survey instrument for school-aged children. *Sleep* **23**, 1043–1051 (2000).

18. Goodlin-Jones, B. L., Sitnick, S. L., Tang, K., Liu, J. & Anders, T. F. The Children's Sleep Habits Questionnaire in toddlers and preschool children. *J. Dev. Behav. Pediatr. JDBP* **29**, 82–88 (2008).

19. Shapiro, E., Potegal, M., Rudser, K. & Delaney, K. Revised Manual for the sanfilippo Behavior Rating Scale (SBRS). (2014).

20. Breau, L. M., McGrath, P. J., Camfield, C. S. & Finley, G. A. Psychometric properties of the non-communicating children's pain checklist-revised. *Pain* **99**, 349–357 (2002).

21. Silva, L. M. T. & Schalock, M. Autism Parenting Stress Index: initial psychometric evidence. *J. Autism Dev. Disord.* **42**, 566–574 (2012).

22. Wikén, M. *et al.* PReS-FINAL-2326: No correlation between anti-drug antibodies and pharmacokinetics, efficacy or safety of Anakinra (Kineret®) in patients with severe CAPS. *Pediatr. Rheumatol. Online J.* **11**, P316 (2013).

23. Ilowite, N. *et al.* Anakinra in the treatment of polyarticular-course juvenile rheumatoid arthritis: safety and preliminary efficacy results of a randomized multicenter study. *Clin. Rheumatol.* **28**, 129–137 (2009).

24. Shapiro, E. G. *et al.* Quantifying behaviors of children with Sanfilippo syndrome: The Sanfilippo Behavior Rating Scale. *Mol. Genet. Metab.* **114**, 594–598 (2015).

25. Breau, L. M. *et al.* Relation between pain and self-injurious behavior in nonverbal children with severe cognitive impairments. *J. Pediatr.* **142**, 498–503 (2003).

26. Carter, A. S. *et al.* The Vineland Adaptive Behavior Scales: Supplementary Norms for Individuals with Autism. *J. Autism Dev. Disord.* **28**, 287–302 (1998).

27. Sparrow, S. S., Cicchetti, D. V. & Saulnier, C. A. *Vineland Adaptive Behavior Scales - Third Edition (Vineland-3)*. (2016).

28. Kowalski, K. & Mulak, A. Brain-Gut-Microbiota Axis in Alzheimer's Disease. *J. Neurogastroenterol. Motil.* **25**, 48–60 (2019).

29. Fattorusso, A., Di Genova, L., Dell'Isola, G. B., Mencaroni, E. & Esposito, S. Autism Spectrum Disorders and the Gut Microbiota. *Nutrients* **11**, (2019).

30. Urien, S. *et al.* Anakinra pharmacokinetics in children and adolescents with systemic-onset juvenile idiopathic arthritis and autoinflammatory syndromes. *BMC Pharmacol. Toxicol.* **14**, 40 (2013).

APPENDIX A: Data and Safety Monitoring Plan (Single-Centered, No DSMB)

Project No. : 031834-01
Protocol Title: Open-label pilot study of the effects of anakinra in mucopolysaccharidosis (MPS) III
Principal Investigator: Lynda Polgreen, MD, MS
Monitor: Richard Vehe, MD, Pediatric Rheumatology, University of Minnesota

**UCLA CTSI:**	☐ UCLA-CHS	☒ LI/ Harbor-UCLA	☐ Cedar-Sinai Medical Center	☐ Charles Drew University
Version:	1.1	Version Date:	17-Apr-2020	

1. **Categorization of risk level (low, medium, or high) and a discussion of the relevant specific considerations.**

Risk Level: ☐ Low ☒ Moderate ☐ High

Justification of Risk Level:
Potential for direct subject benefit.

2. **Specific information to be provided at the time of each periodic review and the bases for selecting this data set.**

Enrollment Data:
The investigator will prepare a periodic report to include target, interval and cumulative data in a tabular format as follows:

- ☒ Number of subjects screened
- ☒ Number of subjects enrolled
- ☒ Number of screen failures with reason for screen failure
- ☐ Number of subjects not qualified for randomization based on screening with reason for screen failure
- ☐ Number of subjects qualified for randomization based on screening
- ☐ Number of subjects randomized
- ☒ Dropout number with reason for dropout and stage of study at dropout
- ☒ Completion number
- ☒ Other study specific enrollment data such as subset enrolled in each group, site, etc. Specify below: enrollment at each site

Adverse Event Reporting:

- ☒ **All study-related adverse events** (including loss of confidentiality and moderate-severe discomfort with study sample collection) with severity and attribution (related or not related).
- ☒ The rates of routine illnesses (URI, headaches, muscle aches, etc.) and traumatic injuries are not expected to be different than usual and will not be reported or tracked as unexpected AEs but will be tracked and reported as AEs for the DSM Reports.
- ☐ The rates of routine illnesses (URI, headaches, muscle aches, etc.) and traumatic injuries are not expected to be different than usual and will **NOT** be reported or tracked as AEs for the DSM Reports.
- ☐ The rates of non-serious adverse event related to ______ and due to routine illnesses (URI, headaches, muscle aches, etc.) and traumatic injuries are not expected to be different than usual and will not be reported or tracked as AEs.
- ☐ **All adverse events** with severity and attribution. [**Note:** This should not be selected unless it is really necessary to report all AEs based on the risk profile of the study.]

Serious Adverse Event and Unexpected Adverse Events:

- ☒ For all study-related serious adverse events (SAEs) and study-related unexpected adverse events (UAEs), the clinical summary will also be provided in a cumulative fashion.
- ☒ Due to the large number of disease-related SAEs such as

  Hospitalization and/or surgery due to seizure, vomiting/diarrhea/dehydration, cervical cord compression, or osteonecrosis of the femoral head. New onset or worsening of visual impairment.

  these serious adverse events will be tracked and evaluated by treatment group at each DSM periodic reporting but will not be reported by expedited reporting to the IRB.
- ☐ For all serious adverse events (SAEs) and all unexpected adverse events (UAEs), the clinical summary will also be provided in a cumulative fashion.

Study Specific Adverse Event Reporting: (If Applicable)

- ☐ Other study specific adverse event reporting. Specify below:

The investigator will comply with the Policies for Reporting of Adverse Events to the local IRB that requires reporting of adverse events that in the opinion of the investigator may represent unanticipated problems involving risks to the other subjects in the research.

- ☒ The investigator will provide a tabulation of the unanticipated problems that meet the IRB reporting requirement with their evaluation of each events in the DSM Report

Monitor's Review:

- ☒ The investigator will provide the report to the Monitor at least 30 day before the CTRC due date for DSM Report submission.
- ☐ All the above data will be provided as blinded data to the independent monitor coded by subject number.
- ☐ The independent monitor will have access to the randomization code if he/she decides it is necessary to determine whether any adverse event or irregularities may be related to study treatment.
- ☐ ____________________ will provide the randomization code to the monitor in order to maintain the blinding of the study. The monitor should specify any portion of the review that should not be seen by the PI or other study personnel due to blinding issues.
- ☐ Other: Specify below.

- ☒ The Monitor will review the report and may request additional information from the investigator as needed to evaluate the safety and progress of the study. The monitor will submit the periodic report to the PI with the following by the due date.

or ☐ The investigator/monitor will submit the periodic report to the CTRC with the following by the due date.

  - ☒ Narrative summary of information presented.
  - ☒ Any concerns regarding adverse event rates, recruitment rates, drop-out rates, study integrity, etc.
  - ☒ Any concerns regarding adverse event rates, recruitment rates, drop-out rates, study integrity, etc. with statement of how these concerns were resolved with the PI.
  - ☒ Recommendations for future study conduct (continuation, modification or termination).

- ☒ The PI will submit the DSM Report with the Monitor's review to the CTRC by the due date via iMedris.

3. Frequency of review, and rationale for recommended frequency.

Frequency of Review: Every 6 months .
Rationale for Review Frequency: Limited number of subjects in the study (20 will be enrolled over 24 months) and the level of moderate risk.

4. Designation of individual(s) as independent monitor(s) reviewing the information. An explicit review of any conflict of interest issues should be presented.

Dr. Richard Vehe will act as the Monitor for this clinical study. Dr. Vehe is a faculty member at the University of Minnesota with an appointment in the Department of Pediatrics, Division of Rheumatology. He is the Director of the Division of Pediatric Rheumatology. He has been a site Investigator for multiple clinical trials for juvenile idiopathic arthritis and other autoinflammatory diseases, including a study of anakinra. He has been a member of multiple DSMB for the NIH. In addition, he has expertise in the clinical use of anakinra.
