# Supplementary material for: Anakinra in Sanfilippo syndrome: a phase 1/2 trial
Source: Nat Med. 2024 Jun 21;30(9):2473–9. doi: 10.1038/s41591-024-03079-3 (PMC11405265; doi:10.1038/s41591-024-03079-3)
Supplement: Supplementary file 2 — Reporting Summary [file 41591_2024_3079_MOESM2_ESM.pdf]

nature portfolio

Corresponding author(s): Lynda Polgreen, MD, MS

Last updated by author(s): May 5, 2024



# Reporting Summary

Nature Portfolio wishes to improve the reproducibility of the work that we publish. This form provides structure for consistency and transparency in reporting. For further information on Nature Portfolio policies, see our Editorial Policies and the Editorial Policy Checklist.

## Statistics

For all statistical analyses, confirm that the following items are present in the figure legend, table legend, main text, or Methods section.

| n/a | Confirmed | |
|---|---|---|
| ☐ | ☒ | The exact sample size (*n*) for each experimental group/condition, given as a discrete number and unit of measurement |
| ☐ | ☒ | A statement on whether measurements were taken from distinct samples or whether the same sample was measured repeatedly |
| ☐ | ☒ | The statistical test(s) used AND whether they are one- or two-sided<br>*Only common tests should be described solely by name; describe more complex techniques in the Methods section.* |
| ☐ | ☒ | A description of all covariates tested |
| ☐ | ☒ | A description of any assumptions or corrections, such as tests of normality and adjustment for multiple comparisons |
| ☐ | ☒ | A full description of the statistical parameters including central tendency (e.g. means) or other basic estimates (e.g. regression coefficient) AND variation (e.g. standard deviation) or associated estimates of uncertainty (e.g. confidence intervals) |
| ☐ | ☒ | For null hypothesis testing, the test statistic (e.g. *F*, *t*, *r*) with confidence intervals, effect sizes, degrees of freedom and *P* value noted<br>*Give P values as exact values whenever suitable.* |
| ☒ | ☐ | For Bayesian analysis, information on the choice of priors and Markov chain Monte Carlo settings |
| ☒ | ☐ | For hierarchical and complex designs, identification of the appropriate level for tests and full reporting of outcomes |
| ☒ | ☐ | Estimates of effect sizes (e.g. Cohen's *d*, Pearson's *r*), indicating how they were calculated |

*Our web collection on statistics for biologists contains articles on many of the points above.*

## Software and code

Policy information about availability of computer code

| | |
|---|---|
| Data collection | Flow cytometry data were acquired using BD FACS Aria III using BD FACSDiva v9.0.1. |
| Data analysis | SAS v9.4 (SAS Institute, Cary, NC); FlowJo v10.8.1; |

For manuscripts utilizing custom algorithms or software that are central to the research but not yet described in published literature, software must be made available to editors and reviewers. We strongly encourage code deposition in a community repository (e.g. GitHub). See the Nature Portfolio guidelines for submitting code & software for further information.

## Data

Policy information about availability of data

All manuscripts must include a data availability statement. This statement should provide the following information, where applicable:

- Accession codes, unique identifiers, or web links for publicly available datasets
- A description of any restrictions on data availability
- For clinical datasets or third party data, please ensure that the statement adheres to our policy

The de-identified individual participant data that underlie the results reported in this article (including text, tables, and figures) are included in Supplementary Data File 1.

## Research involving human participants, their data, or biological material

Policy information about studies with human participants or human data. See also policy information about sex, gender (identity/presentation), and sexual orientation and race, ethnicity and racism.

| | |
|---|---|
| Reporting on sex and gender | Participant sex was collected from the parent/caregiver. Gender data was not collected. This is due to the severe cognitive deficits in our study population such that they are unable to self report sex or gender. Sex and/or gender were not considered in the study design. |
| Reporting on race, ethnicity, or other socially relevant groupings | Participant race and ethnicity was collected from the parent/caregiver using the NIH reporting categories. Participants were unable to self-report due to cognitive deficits.<br>Race and ethnicity was not included in our statistical analysis due to our sample size. Race and ethnicity are reported in Table 1. |
| Population characteristics | These are listed in Table 1. They are age, race, ethnicity, sex, type of MPS III, past experimental therapy with gene therapy or enzyme replacement therapy, and adaptive behavior level using the Vineland III. |
| Recruitment | Participants were recruited through clinicaltrials.gov, the Cure Sanfilippo Foundation website, Social Media, and word of mouth. This may bias against individuals who do not access the Internet. |
| Ethics oversight | The John F. Wolf, M.D. Human Subjects Committee at the Lundquist Institute for Biomedical Innovation at the Harbor-UCLA Medical Center |

Note that full information on the approval of the study protocol must also be provided in the manuscript.

# Field-specific reporting

Please select the one below that is the best fit for your research. If you are not sure, read the appropriate sections before making your selection.

☒ Life sciences ☐ Behavioural & social sciences ☐ Ecological, evolutionary & environmental sciences

For a reference copy of the document with all sections, see nature.com/documents/nr-reporting-summary-flat.pdf

# Life sciences study design

All studies must disclose on these points even when the disclosure is negative.

| | |
|---|---|
| Sample size | In designing our study, we initially chose a sample size of 20 participants based on considerations of feasibility, including resource constraints and the availability of participants with this rare disease. Our primary focus was on ensuring that the study could be completed within our budget and reasonable time-frame, while still maintaining the integrity of our research question. Now that the study is completed, we are pleased to report both clinically and statistically significant results despite the relatively small sample size. |
| Data exclusions | No data were excluded. |
| Replication | All surveys were completed at both screening and 8 weeks later prior to the first dose of study drug (anakinra). There were no statistically significant differences between these two timepoints. |
| Randomization | This was a Phase 1/2 open-label study so no randomization was done. Due to the rare nature of the disease and thus small sample size, no potential covariates were controlled for. |
| Blinding | N/A - open-label study |

# Reporting for specific materials, systems and methods

We require information from authors about some types of materials, experimental systems and methods used in many studies. Here, indicate whether each material, system or method listed is relevant to your study. If you are not sure if a list item applies to your research, read the appropriate section before selecting a response.

## Materials & experimental systems

| n/a | Involved in the study |
|---|---|
| ☐ | ☒ Antibodies |
| ☒ | ☐ Eukaryotic cell lines |
| ☒ | ☐ Palaeontology and archaeology |
| ☒ | ☐ Animals and other organisms |
| ☐ | ☒ Clinical data |
| ☒ | ☐ Dual use research of concern |
| ☒ | ☐ Plants |

## Methods

| n/a | Involved in the study |
|---|---|
| ☒ | ☐ ChIP-seq |
| ☐ | ☒ Flow cytometry |
| ☒ | ☐ MRI-based neuroimaging |

# Antibodies

**Antibodies used**

Identification of blood cell subtypes was performed as follows: briefly, monocytes were gated within the myeloid cell population identified by excluding B cells (CD19-APC-Cy7 positive cells BD cat#561743) and T cells (CD3-PE positive cells Fisher Scientific Cat #555333) from the whole blood population. Within the myeloid population, natural killer cells (NK cells) were excluded using CD56 positive cells (CD56-FITC+ and CD14-Alexa-647- cells cat#340410 and 562690 respectively). Subsequently, neutrophils were identified as bright CD16-PerCP-Cy5.5+ (BD cat# 560717) high side scatter cells (SSC). Cells that were not bright CD16 (neutrophils) in the FSC plot were identified as monocyte. These cells expressed CD14 and CD16. CD4-APC-Cy7+ cells (BD cat # 566319) and CD8-PE-Cy7+ cells (BD cat# 335787) were gated within the CD3+ cells of the whole blood, excluding B cells (CD19-APC+ cells BD cat# 555415), and B cells were gated as CD19+ cells.

List of antibodies
Antibody Clone Fluorophore Cat.no.
CD3 UCHT1 PE 555333
CD4 SK3 APC-Cy7 341095
CD8 SK1 PE-Cy7 335787
CD14 MOP9 Alexa-Fluor 647 562690
CD16 3G8 PerCP-Cy 5.5 560717
CD19 SJ25C1 APC-Cy7 557791
CD19 HIB19 APC 555415
CD56 NCAM16.2 FITC 340410

The antibodies were diluted at a ratio of 1.5:200 and then titrated against blood samples to assess reactivity and binding efficiency.

**Validation**

All antibodies were commercially available and were applied according to the manufacturers' instructions. Validation was performed per DIN EN ISO 15189 criteria.

# Clinical data

Policy information about clinical studies
All manuscripts should comply with the ICMJE guidelines for publication of clinical research and a completed CONSORT checklist must be included with all submissions.

**Clinical trial registration**

NCT04018755

**Study protocol**

Included with submission

**Data collection**

Data was collected from January 2020 through March 2023 at a single site, The Lundquist Institute at Harbor-UCLA Medical Center and via telemedicine visits conducted by the PI and sub-I.

**Outcomes**

All primary and secondary study endpoints were defined according to the aim of the study and prespecified in the clinical protocol.

Primary endpoints were:
Phase 1: Incidence of treatment-emergent adverse events and laboratory values over 8 weeks of treatment to known frequency of anakinra-related AEs in other populations.
Phase 2: Need for dose escalation as determined by within individual change over 8-week treatment period compared to change over 8-week observational period in the 2 most bothersome symptoms for each enrolled patient, selected from measures for the MDRI.

Secondary endpoints included
- A MDRI comprised of:
  1. Sanfilippo Behavior Rating Scale (SBRS)
  2. Child Sleep Health Questionnaire (CSHQ)
  3. Autism Parenting Stress Index (APSI)
  4. PROMIS Fatigue - Parent Proxy Custom Short Form
  5. Movement disorder (e.g. dystonia, chorea, etc.): Parent reported duration and severity.
  6. Non-communicating Children’s Pain Checklist-Revised (NCCPC-R)
- Individual Clinical Response (ICR) – 5 most impactful clinical problems reported by the caregiver. Sum using 5-point Likert scale for

each ICR element.

Post hoc analysis was done for immunophenotype and correlation of this with change in the secondary outcomes included in the MDRI.

# Plants

Seed stocks

*Report on the source of all seed stocks or other plant material used. If applicable, state the seed stock centre and catalogue number. If plant specimens were collected from the field, describe the collection location, date and sampling procedures.*

Novel plant genotypes

*Describe the methods by which all novel plant genotypes were produced. This includes those generated by transgenic approaches, gene editing, chemical/radiation-based mutagenesis and hybridization. For transgenic lines, describe the transformation method, the number of independent lines analyzed and the generation upon which experiments were performed. For gene-edited lines, describe the editor used, the endogenous sequence targeted for editing, the targeting guide RNA sequence (if applicable) and how the editor was applied.*

Authentication

*Describe any authentication procedures for each seed stock used or novel genotype generated. Describe any experiments used to assess the effect of a mutation and, where applicable, how potential secondary effects (e.g. second site T-DNA insertions, mosiacism, off-target gene editing) were examined.*

# Flow Cytometry

## Plots

Confirm that:

- [x] The axis labels state the marker and fluorochrome used (e.g. CD4-FITC).
- [x] The axis scales are clearly visible. Include numbers along axes only for bottom left plot of group (a 'group' is an analysis of identical markers).
- [x] All plots are contour plots with outliers or pseudocolor plots.
- [x] A numerical value for number of cells or percentage (with statistics) is provided.

## Methodology

Sample preparation

Fresh whole blood sample collected in CPT tube was washed twice in PBS and then processed for antibody staining (please see Antibody section). When staining procedure was finished, we acquired 50,000 events on the live cells.

Instrument

BD FACS Aria III

Software

Sample acquisition and recording was performed using FACS DIVA software v9.0.1. Post recording analysis was performed by using FlowJO software version 10.8.1

Cell population abundance

Figure 4 shows an example of the cell population abundance and gating strategy for how the data were analyzed.

Gating strategy

Fragments and debries were excluded in FSC vs SSC scatter plot. Doublets of cells were eliminated by plotting SSC-A vs SSC-H. Live cells were identified using the fixable cell viability dye (BD Cat# BDB564996). Identification of blood cell subtypes was performed as follows: briefly, monocytes were gated within the myeloid cell population identified by excluding B cells (CD19-APC-Cy7 positive cells BD cat#561743) and T cells (CD3-PE positive cells Fisher Scientific Cat #555333) from the whole blood population. Within the myeloid population, natural killer cells (NK cells) were excluded using CD56 positive cells (CD56-FITC+ and CD14- Alexa-647- cells cat#340410 and 562690 respectively). Subsequently, neutrophils were identified as bright CD16-PerCP-Cy5.5+ (BD cat# 560717) high side scatter cells (SSC). Cells that were not bright CD16 (neutrophils) in the FSC plot were identified as monocyte. These cells expressed CD14 and CD16. CD4-APC-Cy7+ cells (BD cat # 566319) and CD8-PE-Cy7+ cells (BD cat# 335787) were gated within the CD3+ cells of the whole blood, excluding B cells (CD19-APC+ cells BD cat# 555415), and B cells were gated as CD19+ cells.

- [x] Tick this box to confirm that a figure exemplifying the gating strategy is provided in the Supplementary Information.
